# A Factor Graph Nested Effects Model To Identify Networks from Genetic Perturbations

**DOI:** 10.1371/journal.pcbi.1000274

**Published:** 2009-01-30

**Authors:** Charles J. Vaske, Carrie House, Truong Luu, Bryan Frank, Chen-Hsiang Yeang, Norman H. Lee, Joshua M. Stuart

**Affiliations:** 1 Biomolecular Engineering Department, University of California Santa Cruz, Santa Cruz, California, United States of America; 2 Department of Pharmacology and Physiology, The George Washington University Medical Center, Washington, D.C., United States of America; 3 Institute of Statistical Science, Academia Sinica, Nankang, Taipei, Taiwan, Republic of China; California Institute of Technology, United States of America

## Abstract

Complex phenotypes such as the transformation of a normal population of cells into cancerous tissue result from a series of molecular triggers gone awry. We describe a method that searches for a genetic network consistent with expression changes observed under the knock-down of a set of genes that share a common role in the cell, such as a disease phenotype. The method extends the *Nested Effects Model* of Markowetz et al. (2005) by using a probabilistic factor graph to search for a network representing interactions among these silenced genes. The method also expands the network by attaching new genes at specific downstream points, providing candidates for subsequent perturbations to further characterize the pathway. We investigated an extension provided by the factor graph approach in which the model distinguishes between inhibitory and stimulatory interactions. We found that the extension yielded significant improvements in recovering the structure of simulated and *Saccharomyces cerevisae* networks. We applied the approach to discover a signaling network among genes involved in a human colon cancer cell invasiveness pathway. The method predicts several genes with new roles in the invasiveness process. We knocked down two genes identified by our approach and found that both knock-downs produce loss of invasive potential in a colon cancer cell line. Nested effects models may be a powerful tool for inferring regulatory connections and genes that operate in normal and disease-related processes.

## Author Summary

Biological processes are the result of the actions and interactions of many genes and the proteins that they encode. Our knowledge of interactions for many biological processes is limited, especially for cancer where genomic alterations may create entirely novel pathways not present in normal tissue. Perturbing gene expression (for example, by deleting a gene) has long been used as a tool in molecular biology to elucidate interactions but is very expensive and labor intensive. The search for new genes that may participate can be a daunting “fishing expedition.” We have devised a tool that automatically infers interactions using high-throughput gene expression data. When a gene is silenced, it causes other genes to be switched on or off, which provide clues about the pathway(s) in which the gene acts. Our method uses the genomewide on/off states as a fingerprint to detect interactions among a set of silenced genes. We were able to elucidate a network of interactions for several genes implicated in metastatic colon cancer. Genes newly connected to the network were found to operate in cancer cell invasion in human cells, validating the approach. Thus, the method enables an efficient discovery of the networks that underlie biological processes such as carcinogenesis.

## Introduction

Carcinogenesis involves a host of cell-cell communication breakdowns that include the loss of contact inhibition, an increased potential to proliferate, and the ability to invade and spread into foreign tissue [1]. The molecular events involved in this transformation are still poorly understood. New systematic methods are needed to infer the key events responsible for these disease processes. The ability to measure gene expression changes for the entire genome in the presence of molecular perturbations, such as specific gene knock-downs, provides a new opportunity to infer gene networks in a data-driven manner.

Our goal is to identify the genetic mechanisms underlying a phenotype, such as cancer cell deregulation. We take a network-based approach to the problem, starting with a set of *signaling genes* or S-genes, known to act in a common pathway. The input to the method is a matrix in which gene expression has been measured under the knock-down of each of the S-genes. Genes exhibiting differential expression across the knock-downs, here referred to as *effect genes* or E-genes, are used to predict a set of interactions among the S-genes, and expand the pathway by identifying newly implicated *frontier genes* based on their expression changes. We hypothesize that using a structured model of the interactions among the S-genes will improve the identification of frontier genes for inclusion in the network for subsequent rounds of investigation.

Previous approaches for pathway expansion have used methods based on expression correlations to a phenotype of interest. These methods search for genes with expression profiles that are highly correlated with a particular phenotype or disease state and have led to promising results [2]–[5]. Methods using Analysis of Variance [6], false-discovery [7], and non-parametric methods [8] also have been proposed. For example, one method is to measure the correlation of gene expression levels with an idealized vector representing the phenotype (e.g. indicator variables with zeroes for disease and ones for lack of disease) [9]. One disadvantage of these methods is that they make no explicit use of the known members of a pathway or how these members interact with each other.

More recently, several approaches have demonstrated learning a structured model from perturbation experiments [10]–[13]. Approaches based on Bayesian Networks have also been proposed [11],[12]. However, these approaches attempt to identify networks over the E-genes rather than the S-genes and therefore require many replicated microarray experiments to distinguish signal from noise. Instead, perturbing genes of interest and constructing networks from observations of downstream changes allows powerful interventional reasoning, as well as reconstruction of interactions not directly reflected in expression levels, such as phosphorylation. In one approach, Carter et al. (2007) [14] decompose the matrix of expression changes under single- and double-gene deletions to infer a transcriptional regulation network from which phenotypes and gene expression responses following knock-downs can be predicted. An alternative approach is the *Nested Effects Model* (NEM) of Markowetz et al. (2005, 2007) [10],[15], which has been used to predict interactions, including non-transcriptional interactions. Rather than searching for genetic networks that explain observational data, as several Bayesian Network approaches have done [11],[16], NEMs are useful in situations in which perturbations have been carried out on a focused set of genes. In this case, NEMs assume the interest is in a finer description of the interactions among the silenced genes rather than identifying a network of unrestricted connections between potentially additional genes. The NEM approach takes as input a matrix of expression changes, *X*. A column of *X* corresponds to a single gene knock-down (or knock-out) of one S-gene; a row corresponds to the response of an E-gene to all of the knock-downs. The method searches for approximate subset relations among the expression changes of the E-genes to organize the S-genes into a network. To do this it assumes, for example, that S-gene *A* is above S-gene *B* if the set of E-genes that change under gene *A*'s knock-down are an approximate superset of the effected genes under *B*'s knock-down.

The current NEM approach uses binary set membership relations to identify a network and thus the exact nature of interaction between S-genes (e.g. activation or inhibition) is not determined. However, an appreciable extent of inhibition occurs in real genetic networks. To estimate the amount of inhibition present in living cells, we estimated the proportion of genes up-regulated in deletion mutants relative to wild-type from a yeast knock-out compendium [17]. Over half of the genes had increasing expression changes across the deletion strains, consistent with a high degree of inhibitory interactions in the yeast genetic network (see Figure S1). Thus, the inability to distinguish between stimulatory and inhibitory interactions may be a critical shortcoming of current NEM approaches.

To address this limitation, we developed a generalization of the NEM approach using a probabilistic graphical model called a factor graph that allows a broader set of S-gene interactions to be recovered from the secondary effects of E-gene expression. This paper offers three methodological contributions. First, we present a factor graph formulation called FG-NEM that allows for an efficient search over all possible NEM structures for a high-scoring model. Second, we show how FG-NEMs extend the NEM approach for expanding the network beyond the current set of S-genes. Third, we show that FG-NEMs can model a more general class of S-gene interactions than NEMs, which increases the accuracy of network identification over an approach that considers a more restricted set of interactions.

We demonstrate the usefulness of FG-NEMs on both simulated and biologically relevant signaling networks that contain both inhibition and activation. We apply FG-NEMs to identify novel genes not previously implicated in colon cancer cell invasiveness. Finally, we experimentally test FG-NEM predictions and report that knock-downs of the top-scoring genes lead to a loss-of-invasion phenotype, validating the approach. Source code is available as an R library from our website: http://sysbio.soe.ucsc.edu/projects/fgnem.

## Methods

We first describe the Nested Effects Model, derive a *maximum a posteriori* objective function to identify highly probable networks, and then describe how to recode the search for a network as inference on a factor graph. We then discuss how we expand the *frontier* of the network by identifying new genes that have high attachment probability using modified NEM attachment scoring. Finally, we describe our method for validating the involvement of these frontier genes using directed knock-down and phenotypic assays.

### The Nested Effects Model

Our goal is to automatically identify genetic interactions among a set of *signaling genes* from gene expression changes observed under their knock-down. The signaling genes represent a set of genes that prior experimental evidence suggests participate in a common pathway. To infer a network, we use an extension of the *Nested Effect Model (NEM)* introduced by Markowetz et al. (2005) [10]. The set of silenced genes are denoted as the set ***S*** (or S-genes). An NEM is a probabilistic formulation that measures how well a directed graph of the S-genes is consistent with expression changes collected under the separate silencing of each S-gene (i.e. only single knock-downs are considered in NEM). While the method can make use of either complete deletion mutants or genes that may be partially silenced, here we use the term knock-down to refer to either case. We denote the knock-down of S-gene *A* as Δ*A*. We also refer to a set of *effect genes* as the set ***E*** (or E-genes), for which gene expression data is available. The expression of an E-gene *e* is assumed to be influenced by at most one S-gene. The key assumption of NEMs is the expression changes observed under Δ*A* are an approximate superset of the changes observed under Δ*B* if gene *A* acts upstream of gene *B* in a pathway. We use the shorthand *A*>*B* to represent this generic directed interaction.

**Figure 1:**
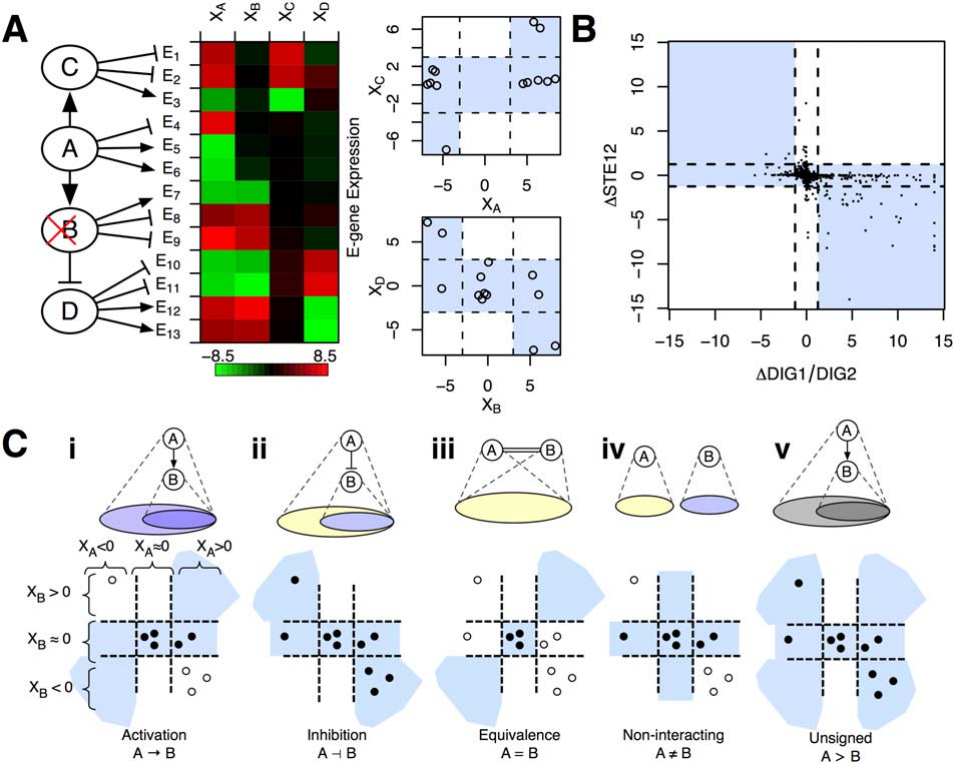
Predicting Pair-wise Interaction Using Quantitative Nested Effects. (A) Hypothetical example with four S-genes, *A*, *B*, *C*, and *D*. The graph contains one inhibitory link, B⊣D (left). A heatmap of E-gene expression under knockdown of each S-gene shows both inhibitory and stimulatory effects (middle). Scatter plots of the *C*, *A*, *B*, and *D* knock-outs show that expression fits in the shaded preferred regions of each interaction (right). The inhibitory link explains some of the “observed” data: expression changes under *ΔD* (bright red or bright green entries in the heatmap) occur in a subset of the E-genes for which the opposite changes occur in *ΔB*. (B) Data from a known inhibitory interaction. Expression levels of effect genes under the DIG1/DIG2 knock-out (*y*-axis) plotted against their levels under the STE2 knock-out (*x*-axis) as detected in [17]. Expression changes significant at *α* = 0.05 indicated in gray lines. DIG1/DIG2 is known to inhibit STE12. (C) Interaction modes. Observed E-gene expression changes are compared to five possible types of interactions between two S-genes, *A* and *B* (i–v). The top row illustrates the expected nested effects relationship for each type of interaction mode: circles represent sets of E-genes with expression changes consistent with either activation (blue circles) or inhibition (yellow circles). Scatter-plots for each interaction mode show the hypothetical expression changes under *ΔA* (*x*-axis) and *ΔB* (*y*-axis) for all E-genes (circles). E-gene levels are either consistent (filled) or inconsistent (open) with the mode. Shaded regions demark expression levels consistent with each interaction model. The example shows expression changes that most closely match the inhibition mode (indicated by the greatest number of closed circles).

In addition to identifying *A*>*B*, the E-gene expression changes on the microarray can be used to infer the “sign” of the interaction, either activating or inhibiting. In our framework, we extend the interactions so that an upstream gene can have either an inhibitory or stimulatory effect on downstream genes. Figure 1A presents an example, similar to Fröhlich et al. (2008) [18] that motivates the use of signed interactions. E-genes *E_1_* through *E_13_* are listed from top to bottom according to where they are attached to the network. Depending on the connections of the S-genes to one another and to the E-genes, a disruption in an S-gene will cause E-genes to either increase or decrease in expression relative to wild-type. For example, E-gene *E_7_* decreases under Δ*B* relative to wild-type because the wild-type activation by *B* is absent in the deletion. On the other hand, the expression of *E_10_* also decreases under Δ*B* relative to wild-type but as a result of a different mechanism. In wild-type, *E_10_* is expressed at a baseline level because its repressor, the product of gene *D*, is inhibited by *B*'s product. However, in the *B* deletion, *D* is derepressed, leading to inhibition of *E_10_*. This toy example illustrates that the disambiguation of inhibition and activation, both for S-gene interactions and E-gene attachments, make it possible to account for an expanded set of mechanisms leading to the observed expression changes.

The E-gene expression changes are available in a data matrix *X* where each column gives the difference in expression of each E-gene under the deletion of a single S-gene relative to wild-type. *X* may also contain replicates in the form of repeated S-gene knock-downs. The entry *X_eAr_* represents *e*'s expression change under the *r* ^th^ replicate of Δ*A*. Furthermore, we assume that an unknown expression “state” for each E-gene under each knock-down, determines its set of expression changes observed across the {*X_eAr_*} replicates in the microarray data. The matrix, *Y*, records a hidden state for each E-gene under each knock-down, where entry *Y_eA_* is the state of E-gene *e* under Δ*A*. We allow the states to be ternary-valued {+1, −1, 0} representing whether *e* is up-regulated, down-regulated, or unchanged under Δ*A* relative to wild-type respectively.

Nested effects models include two sets of parameters. The parameter set *Φ* records all pair-wise interactions among the S-genes and the parameter set *Θ* describes how each E-gene is attached to the network of S-genes. In the original NEM formulations [10],[15],[18] *Φ* is a binary matrix with entry *φ_AB_* set to one if S-gene *A* acts above S-gene *B* and zero otherwise. If *φ_AB_* = *φ_BA_* = *1* then the S-genes are assumed to operate at an equivalent position in the pathway. Note that indirect interactions are also represented in *Φ* so that if *φ_AB_* = *1* and *φ_BC_* = *1* it implies *φ_AC_* = *1*. A parsimonious network among the S-genes is solved for by computing the transitive reduction of *Φ*.

To allow for both stimulatory and inhibitory interactions in our formulation, *φ_AB_* can assume six possible values for each unique unordered S-gene pair {*A*, *B*}. We refer to these values as *interaction modes*. The possible values are: (i) *A* activates *B*, *A*→*B*; (ii) *A* inhibits *B*, *A*⊣*B*; (iii) *A* is equivalent to *B*, *A* = *B*; (iv) *A* does not interact with *B*, *A*≠*B*; (v) *B* activates *A*, *B*→*A*; and (vi) *B* inhibits *A*, *B*⊣*A*.

Plotting the response of E-genes under Δ*A* and Δ*B* yields a scatter-plot that may provide a signature for the type of interaction between *A* and *B*. For example, Figure 1B shows a scatter-plot of gene expression changes from the Hughes et al. (2000) yeast knock-out compendium [17] for a pair of knock-outs of the well-known pheromone-response genes: ΔSTE12 and the ΔDIG1/DIG2 double knock-out. Comparing the scatter-plot for these pheromone-response genes to the patterns in Figure 1C, it can be seen to match the inhibitory interaction mode more closely than the other modes, which is consistent with DIG1/DIG2's known inhibition of STE12. Figure 1C shows an example of the first four modes. Shaded regions denote consistent E-gene responses for each mode. An interaction mode determines a constraint on the observed E-gene expression changes. For example, plotting the expression changes of E-genes that act downstream of either *A* or *B* for the generic *A*>*B* interaction mode produces points in one of the seven shaded regions shown in Figure 1Cv. Figure 1Cii shows an example where the inhibitory interaction mode is the best match to the data because a higher number of E-gene changes fall within consistent regions (filled circles in the figure). In this manner, genomewide expression changes detected on the microarrays can be used as quantitative phenotypes to identify a variety of interactions between pairs of S-genes.

Note that two genes are equivalent if their knock-downs lead to significantly similar expression changes, which may predict, for example, that they form a complex. Figure 1C also illustrates the generic interaction mode *A*>*B* used in an unsigned version of our method. We compare FG-NEM results to two unsigned variants to estimate the change in predictive power as a function of the introduction of sign. In effect, both variants consider four interaction modes: (i) *A*>*B*; (ii) *B*>*A*; (iii) *A*≠*B*; and *A* = *B*. For comparison purposes, a predicted unsigned interaction was treated as activation. In the FG-NEM AVT variant, FG-NEM is run on the absolute value of the data. In the uFG-NEM method, we remove the component of FG-NEM which models induced expression, resulting in interaction modes where the top and right five regions are disallowed in all interaction modes.

### Probabilistic Formulation of NEMs

Our goal is to find a structure among the S-genes that provides a compact description of *X*. To find a network that best “fits” the data, we take a *maximum a posteriori* approach as in [15],[18] jointly identify *Φ* and *Θ* that maximize the posterior:
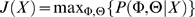
(1)

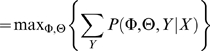
(2)where we introduce the hidden E-gene states by summing over all possible configurations of the *Y* matrix. Applying Bayes' Rule and dropping *P(X)*, which is constant with respect to the maximization, we obtain:
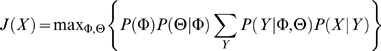
(3)

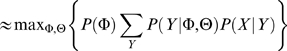
(4)The approximation in the last step uses the assumption that any E-gene attachments are equally likely given a network structure; i.e. *P(Θ|Φ)* is assumed to be uniformly distributed and is ignored in our approach. *P(Φ)* represents a prior over S-gene networks.

As in previous NEM formulations, we assume that each E-gene is attached to a single S-gene and that each E-gene observation vector across the knock-downs is independent of other E-gene observations. The maximization function can then be written:
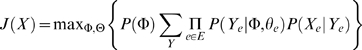
(5)

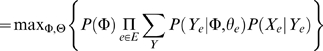
(6)

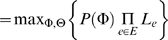
(7)where *X_e_* and *Y_e_* are the row vectors of data and hidden states for E-gene *e* respectively, and *θ_e_* records the attachment point information for E-gene *e*. After rearranging the products and sums, we introduce the shorthand *L_e_* to represent the likelihood of the data restricted only to E-gene *e*.

Previous approaches decompose *L_e_* over the knock-downs, which assume the S-gene observations are independent given the network and attachments (see [18] for an example of such a derivation). To facilitate scoring the expanded set of interaction modes mentioned earlier, we replace *L_e_* with a function proportional to *L_e_*, *L_e_′*. *L_e_′* is defined as a product of pair-wise S-gene terms:
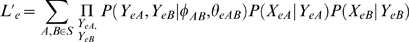
(8)where *θ_eAB_* represents the attachment of E-gene *e* relative to the pair of S-genes *A* and *B*. Note that both *θ_eAB_* and *φ_AB_* are indexed by the unordered pair, {*A*, *B*}, so that *φ_AB_* and *φ_BA_* are references for the same variable. We refer to *θ_eAB_* as *e*'s *local attachment* which can take on five possible values from the set {*A*, *−A*, *B*, *−B*, *0*} representing that *e* is either up- or down-regulated by *A*, attached and either up- or down-regulated by *B*, or not affected by either S-gene. *φ_AB_* defines the mode of interaction between S-genes *A* and *B*. Assuming the replicates are independent given the E-gene states, *P(X_eA_ | Y_eA_)* can be written as a product over replicate terms: 
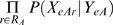
, where *P(X_eAr_ | Y_eA_)* is modeled with a Gaussian distribution having mean 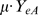 and standard deviation *σ* estimated from the data (see Text S1).

Substituting *L_e_′* for *L_e_* into Eq. (7) and distributing the maximization over attachment points, we obtain the maximizing function used in our approach:
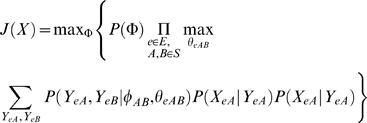
(9)The interaction factors *P*(*Y_eA_*, *Y_eB_* | *φ_AB_*, *θ_eAB_*) have a value of one if the E-gene *e* is attached to either *A* or *B* and *e*'s state is consistent with the interaction mode between *A* and *B*. If *e*'s state is inconsistent with the interaction and attachment, then the factor has value zero. While we used hard constraints to model consistent and inconsistent expression changes (corresponding to the rigid boundaries of the regions drawn in Figure 1C), such constraints could be softened to use factors with belief potentials between zero and one. Note that, to simplify the example, the interaction modes in Figure 1C show defined regions. However, *P(X_eA_ | Y_eA_)* is modeled as a Gaussian distribution and therefore assigns non-zero probabilities over all possible expression values rather than classifying some as allowed and others disallowed (i.e. probability zero).

### An Interaction Transitivity Prior

The prior over interactions, *P(Φ)*, can represent preferences over specific interactions in the S-gene graph, allowing the incorporation of biologically-motivated constraints to guide network search. For example, the interaction priors for genes in a common pathway or genes whose products have been detected to interact in protein-protein interaction screens could be set higher than the priors for arbitrary pairs of S-genes. In this study, we chose to test the approach both with and without external biological information. Without external biological information, the prior encodes a basic property of the S-gene graph: that it should exhibit transitivity to force pair-wise interaction modes to be consistent among all triples. Using transitivity, all paths between any two genes, *A* and *B*, are guaranteed to have the same overall effect; i.e. the product of the signs of individual links along different paths between *A* and *B* are equal.

In order to preserve the transitivity of identified interaction modes, the prior is decomposed over interaction configurations into transitivity constraints on all triples of S-genes; i.e.:
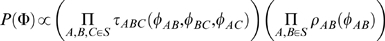
(10)where τ is zero if the triple of interactions are intransitive, and one if the interactions are transitive (see Text S1 for full definition). Using transitivity constraints forces the search to find consistent models that best explain the observed changes. The transitivity constraint includes both the direction of interactions and the sign of interactions. As S-gene interactions are signed, the transitivity constraint forces the sign of the product of two edges to equal the sign of the third; e.g. if *A*⊣*B* and *B*⊣*C*, then *A*→*C*. A result of modeling transitivity is that a directed cycle of stimulatory interactions will also imply activation between any pair of S-genes in the cycle, in both directions. Therefore, the method clusters such S-genes into equivalence interactions. The product over *ρ* factors in Eq. (10) encode evidence from high-throughput assays, such as protein-protein binding and protein-DNA binding interactions (see “Physical Structure Priors” in Text S1).

While network structures are constrained to reflect more intuitive models, the decomposition introduces interdependencies among the interactions, adding complexity to the search for high-scoring networks. Importantly, max-sum message passing in a factor graph [19] provides an efficient means for estimating highly probable S-gene configurations. We next describe how the problem is recoded into message-passing on a factor graph.

### Inference on Factor Graphs to Search for Candidate S-Gene Networks

The formulation above provides a definition of the objective function to be maximized but says nothing about how to search for a good network. The search space of networks is very large making exhaustive search [10] intractable for networks larger than five S-genes. To apply the method to larger networks, we require a fast, heuristic approach. Markowetz et al. (2007) introduced a bottom-up technique to infer an S-gene graph. They identify sub-graphs of S-genes (pairs and triples) and then merge the sub-graphs together into a final parsimonious graph. Fröhlich et al. (2008) [18] use hierarchical clustering to first identify *modules*, subsets of S-genes with correlated expression changes. Networks among the modules are exhaustively searched and a final network is identified by greedily introducing interactions across modules that increase the likelihood.

Here, we introduce the use of a graphical model called a factor graph to represent all possible NEM structures simultaneously. The parameters that determine the S-gene interactions, *Φ*, are explicitly represented as variables in the factor graph. Identifying a high-scoring S-gene network is therefore converted to the task of identifying likely assignments of the *Φ* variables in the factor graph. A factor graph is a probabilistic graphical model whose likelihood function can be factorized into smaller terms (factors) representing local constraints or valuations on a set of random variables. Other graphical models, such as Bayesian networks and Markov random fields, have straightforward factor graph analogs. A factor graph can be represented as an undirected, bi-partite graph with two types of nodes: variables and factors. A variable is adjacent to a factor if the variable appears as an argument of the factor. Factor graphs generalize probability mass functions as the joint likelihood function requires no normalization and the factors need not be conditional probabilities. Each factor encodes a local constraint pertaining to a few variables.

### The Factor Graph for Nested Effects

**Figure 2:**
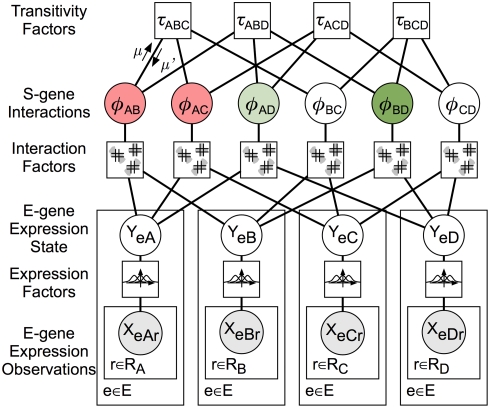
Structure of the factor graph for network inference. The factor graph consists of three classes of variables (circles) and three classes of factors (squares). *X_eAr_* is a continuous observation of E-gene *e*'s expression under *ΔA* and replicate *r*. *Y_eA_* is the hidden state of E-gene *e* under *ΔA*, and is a discrete variable with domain {*up*, ∅, *down*}. *φ_AB_* is the interaction between two S-genes *A* and *B*. Expression Factors model expression as a mixture of Gaussian distributions. Interaction Factors constrain E-gene states to the allowed regions shown in Figure 1C. Transitivity Factors constrain pair-wise interactions to form consistent triangles. The arrows labeled μ and μ′ are messages encoding local belief potentials on *φ_AB_* and are propagated during factor graph inference.

Figure 2 shows the factor graph representing the NEM for the example S-gene network from Figure 1A. Each random variable is represented by a circle and each conditional probability term in Eqs. (9–10) is represented by a square. The factor graph contains three types of variables. First, every unique unordered pair of S-genes *{A,B}* has a corresponding variable, *φ_AB_*, that takes on values equal to one of the previously mentioned interaction modes (Figure 2, “S-Gene Interactions” level). Second, every E-gene-S-gene pair is associated with a variable, *Y_eA_* for the hidden expression state of effect gene *e* under knock-down *A*, (Figure 2, “E-gene Expression State” level). Third, every observed expression value is associated with a continuous variable, *X_eAr_*, where *r* indexes over replications of *ΔA* (Figure 2, “E-gene Expression Observation” level). Figure 2 also shows the expression factors, interaction factors, and transitivity factors of Eqs. (9–10).

#### Inference with message passing

The *Φ* that maximizes the posterior is found using max-sum message passing using all terms from Eqs. (9–10) in log space. For acyclic graphs, the marginal, max-marginal and conditional probabilities of single or multiple variables can be exactly calculated by the max-sum algorithms [19]. Message-passing algorithms demonstrate excellent empirical results in various practical problems even on graphs containing cycles such as feed-forward and feed-back loops [20]–[23].

Here, the message passing schedule performs inference in two steps. In the first step, messages from observations nodes *X_eAr_* are passed through the expression factors and hidden E-gene state variables, to calculate all messages *μ(Y_A_→φ_AB_)* in a single upward pass. In the second step, messages are passed between only the interaction variables and transitivity factors until convergence (see Text S1). In the example shown in Figure 2, running inference results in assignments of activation for *φ_AB_* and *φ_BC_* (shaded red), inhibition for *φ_BD_* and *φ_AD_* (shaded green), and non-interaction for *φ_AB_* and *φ_BC_* (unshaded), which match the NEM structure from Figure 1A. For display of inferred S-gene networks, we compute the transitive reduction of *Φ* by removing all links for which there is a longer redundant path [24].

#### Pathway expansion with FG-NEMs

Once a signaling network is identified using the message passing inference procedure above, the network can be used to search for new genes that may be part of the pathway. The NEM and FG-NEM framework predict new members that act in the pathway by “attaching” E-genes to S-genes in the network, or leaving them detached if their expression data does not fit the model. Attaching E-gene, *e*, to S-gene, *s*, asserts that the expression changes of *e* over all knock-downs are best explained by a network in which *e* is directly downstream of *s*. The E-genes attached to the network are collectively referred to as the *frontier*. Frontier genes may be good candidates for further characterization (e.g. knock-down and expression profiling) in subsequent experiments.

To gain a global picture for where *e* is connected, we use a modified NEM scoring from Markowetz et al. (2005). The pair-wise attachments for a single E-gene connection variable *θ_eAB_*, provide local “best guesses” for *e*'s attachment. Rather than aggregate *e*'s collection of local attachments, we use NEM scoring, modified to incorporate both stimulatory and inhibitory attachments, to estimate the attachment point using the full network learned in the previous step (see Text S1).

We calculate a log-likelihood ratio that measures the degree to which *e*'s expression data is explained by the network if it is attached to one of the S-genes compared to being disconnected from the network, i.e. its likelihood was generated entirely by the background Gaussian distribution. For E-gene *e*, we compute the log-likelihood of attachment ratio (LAR):
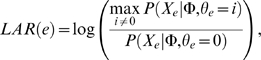
where *θ_e_* here represents Markowetz et. al's attachment parameter expanded to include inhibitory and stimulatory attachments. We rank all of the E-genes according to their LAR scores. Top-scoring genes have data that is more likely to have arisen from the model than a null background. Any E-gene that has a positive LAR score is included as a frontier gene.

### Experimental Validation Procedure for Newly Predicted Cancer Invasion Genes

To validate the involvement of predicted invasiveness frontier genes, HT29 colon cancer cells were resuspended in DMEM medium containing 0.1% FBS and seeded into the top wells (2×10^5^ per well) containing individual Matrigel inserts (BD Biosciences, San Jose, CA) according to manufacturer's protocol. The lower wells were filled with 800 µl medium with 10% fetal bovine serum as chemoattractant. Six to ten hours following seeding, the cells in the upper wells were transfected with the appropriate shRNA-expressing pSuper constructs [25] using Lipofectamine 2000 (Invitrogen, Carlsbad, CA). Final concentration of pSuper constructs was 1.6 µg/ml. The transfected cells were incubated at 37°C for 48 hours before assaying for invasion. Media was aspirated from the top wells and non-invading cells were scraped from the upper side of the inserts with a cotton swab and invading cells on the lower side were fixed and stained using DiffQuick (IMEB, Inc. San Marcos, CA). Total number of invading cells was counted for each insert using a light microscope. Invasion was assessed in quadruplicate and independently repeated at least five times. The shRNA-expressing portion of the construct was designed using the siRNA Selection Program of the Whitehead Institute for Biomedical Research (http://jura.wi.mit.edu/bioc/siRNAext/), synthesized by Invitrogen and subcloned into the XhoI and BamHI sites of pSuper plasmid. Sequences for shRNA constructs are available in the Text S1. shRNA construct MYO1G targets the myosin 1G mRNA (GenBank accession number NM _033054). shRNA construct BMPR1A targets the bone morphogenetic protein receptor, type IA mRNA (NM_004329). shRNA construct COLEC12 targets the collectin sub-family member 12 mRNA (NM_130386). shRNA construct AA099748 targets an expressed sequence tag mRNA (AA099748). shRNA construct CAPN12 targets the calpain 12 mRNA (NM_144691). shRNA construct scrambled serves as a nonsense sequence negative control.

## Results

### Results on Artificial Networks

#### Data

We evaluated FG-NEMs ability to recover artificial networks from simulated data. Data was generated by propagating signals in networks containing simulated knock-downs and then sampling expression data from activated, inhibited, or unaffected expression change distributions (see Text S1 and Figure S3). We focused on how the FG-NEM approach increased recovery of networks that contain both activation and inhibition. Because FG-NEMs explicitly incorporate inhibition, we hypothesized that they would recover networks containing an appreciable amount of inhibition more accurately than an approach lacking separate modes for inhibition and activation. We implemented a version of FG-NEM in which inhibition encoded in the FG-NEM model was removed (see Methods). We refer to this version as the “unsigned” FG-NEM (uFG-NEM). We compared uFG-NEM to the original NEM approach and found that the results were comparable on small synthetic networks of four S-genes and their associated data (see Figure S2). We therefore used uFG-NEMs as a surrogate for NEMs for the tests on larger networks on which NEM was not efficient enough to run.

To make the comparison of FG-NEM to uFG-NEM fair, we measured network recovery in two ways. 1) We calculated a measure of *structure recovery*: a predicted interaction was called correct if it matched an interaction (of either sign) in the simulated network. In this case, whether the interaction was inhibitory or stimulatory was ignored. 2) We measured *sign recovery*: a predicted interaction was recorded as correct if it matched an interaction in the simulated network and had the matching sign.

#### Influence of inhibition extent on network recovery

**Figure 3:**
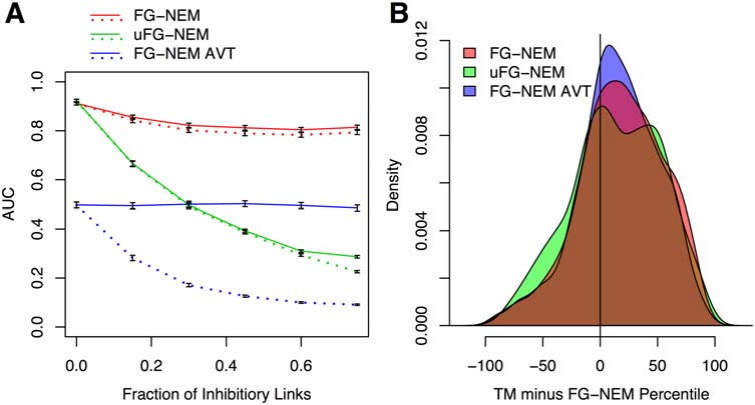
Accuracy of artificial network recovery and expansion. (A) Influence of inhibition on network recovery. AUC (*y*-axis) plotted as a function of the percent of inhibitory links (*x*-axis). Four replicate hybridizations were used in all simulations. Points and error bars represent means and standard deviations computed across 500 synthetically generated networks respectively. Lines in each plot represent the performance of FG-NEM (red) and uFG-NEM run on the original data (green) or on AVT data (blue) for both structure recovery (solid lines) and sign recovery (dotted lines). (B) Accuracy of FG-NEM network expansion compared to Template Matching. The percentile of an S-gene obtained from Template Matching was subtracted from the percentile of the LAR score (see Methods) assigned by FG-NEM and uFG-NEM obtained from the leave-one-out expansion test. A smoothed histogram for FG-NEM (red), uFG-NEM run on the original data (green) and the AVT data (blue) was plotted and shows the proportion of S-genes (*y*-axis) with a particular difference in method percentile (*x*-axis). The underlying simulated network had 32 S-genes, eight S-genes were used for network recovery, and twenty E-genes were attached to each S-gene.

We tested the ability of FG-NEMs and uFG-NEMs to recover the structure of networks simulated with varying fractions of inhibition, 0≤*λ*≤0.75, for both the amount of inhibitory connections between S-genes and inhibitory attachments of E-genes. We simulated and predicted 500 networks, calculated the area under the precision-recall curve (AUC) for each predicted network (see Text S1), and recorded the mean and standard deviation of these AUCs. As expected, when no inhibition was present, FG-NEM and uFG-NEM were equivalent in terms of AUC when run on non-transformed data (Figure 3A). Surprisingly, FG-NEM run on the AVT data performs much worse than FG-NEM even with no inhibition. This may be due to its interpretation of unaffected E-gene changes as affected changes which adds noise to its estimates of hierarchical nesting. As increasing amounts of inhibition is added into simulated networks, the performance of uFG-NEM degrades precipitously for structure recovery, underperforming FG-NEM by a margin of more than 0.20 units of AUC at the highest levels of simulated inhibition (Figure 3A). Even at moderate levels of inhibition, for example at the 15% inhibition level, FG-NEM's AUC is already significantly higher than uFG-NEM's AUC. We also calculated the AUC for recovering the correct sign of the interactions for the unsigned models. In this case, unsigned interactions were interpreted to be activating interactions. As expected, the AUC decreases quadratically since both the precision and recall decrease linearly with increasing fraction of inhibition. Given these results, we expect FG-NEMs to have significantly better performance on real genetic networks where appreciable amounts of inhibition exist (see Figure S1). We also varied other simulation parameters and found that including sign in the model enables FG-NEMs to retain its high level of accuracy in network recovery using fewer microarray replicates and lower proportions of genes from the true network as S-genes (see Text S1 and Figure S4B).

We repeated the experiment of varying inhibition to match our expectations for application to the cancer invasion network discussed subsequently. In the invasion network the known S-genes were recovered in such a way that only activating S-gene connections were identified. To simulate this situation, we created networks containing only activating S-gene interactions but varied the proportion of inhibiting E-gene attachments. Even in this situation where all of the known S-genes have activating interactions, FG-NEM's performance begins to significantly surpass uFG-NEM's performance when 40–60% of the E-genes are connected with inhibitory attachments (see Figure S4C). Thus, according to our simulations, even in cases where activation predominates the S-gene interactions, incorporating sign in the model for E-gene changes can lead to higher network recovery accuracies. We expect the signed FG-NEM to also perform well for the invasion network where 40–60% of the expression changes are consistent with inhibited E-gene attachments.

#### Expansion of artificial networks compared to Template Matching

Because our goal is to elucidate the network of genes involved in the colon cancer invasiveness pathway, we measured the ability of our method to expand the network to new genes involved in the pathway compared to a correlation-based method we refer to as Template Matching (TM) used by Irby et al. (2005) [26]. Briefly, Template Matching [9] ranks genes based on the correlation of their expression profiles to an idealized profile/template that reflects a phenotype of interest. TM has been used in several studies to identify genes with expression patterns that follow a series of phenotypes [27],[28]. We found that FG-NEMs significantly outperform TM when used to expand artificial networks (Figure 3B). We compared TM with FG-NEM using a leave-one-out test in which knock-down data from one S-gene was removed from the dataset (see Text S1). We found that both FG-NEM and uFG-NEM rank a held-out signaling gene higher than TM on average. This is evident in Figure 3B in which all three distributions of LAR percentile differences are shifted to the right of zero. On average, FG-NEM predicts a held-out S-gene 25.3 (+/−15) percentile units higher than TM.

### Network Expansion on a Yeast Knock-Out Expression Compendium

We hypothesized that an estimate of genetic pathway structure based on modeling observed expression changes could facilitate the identification of new pathway members. To test this, we evaluated the ability of FG-NEMs, uFG-NEMs, and TM to identify genes involved in a diverse set of pathways in *S. cerevisae* using the well-studied gene expression dataset from the Hughes et al. (2000) knock-out compendium elucidated by Rosetta [17]. This compendium contains whole-genome expression profiles of 276 yeast gene-deletion mutants and *P* values for differential gene expression.

#### Data

In each deletion strain, gene expression changes with a *p*-value smaller than 0.05 were selected, and then labeled as activated or inhibited according to the sign of their expression log-ratio. *p*-values were converted to continuous expression values using the method of Yeang et al. (2004) [13]. The method replaces a *p*-value with a value obtained by inverting a Chi-square distribution. The value can be interpreted as a log-likelihood ratio reflecting the probability that an E-gene is expressed in the affected distribution compared to a background distribution. Gene sets, representing proxies for pathways, were taken from Gene Ontology (GO) [29], KEGG [30] and Reactome [31] information. 25 non-redundant pathways were selected that had at least 5 genes included as knock-outs in the knock-out compendium. The largest pathway, chromosome organization and biogenesis, contained 45 S-genes. On a 2.83 GHz processor, factor graph inference using 5046 E-genes took a total of 1828 seconds. A pathway with 12 genes, such as nitrogen compound metabolism, took 38 seconds for network inference.

The factor graph approach allows prior information to be incorporated. We tested a supervised variant of FG-NEMs (sFG-NEM) in which additional factors were incorporated to reward models that included known interactions. Three classes of physical data were downloaded for use as interaction priors: protein-DNA interactions, phosphorylation target data, and protein-protein interactions (PPI). Protein-DNA interactions with a p-value less than 0.001 were selected from the study of Lee et al. (2002) [32]. Data describing kinase targets was taken from the study of Ptacek et al. (2005) [33]. PPI data was downloaded from the BioGRID database [34] on July 30, 2008. For each GO category under study, we selected any interaction between S-genes in that category, resulting in 27 Protein-DNA interactions, 4 phosphorylation interactions, and 64 PPIs for the GO sets discussed in this paper. For each unique physical interaction, we added an additional factor to the corresponding interaction variable to increase the likelihood of consistent interaction modes and decrease the likelihood of inconsistent modes (see Text S1).

#### Pathway expansion performance

**Figure 4:**
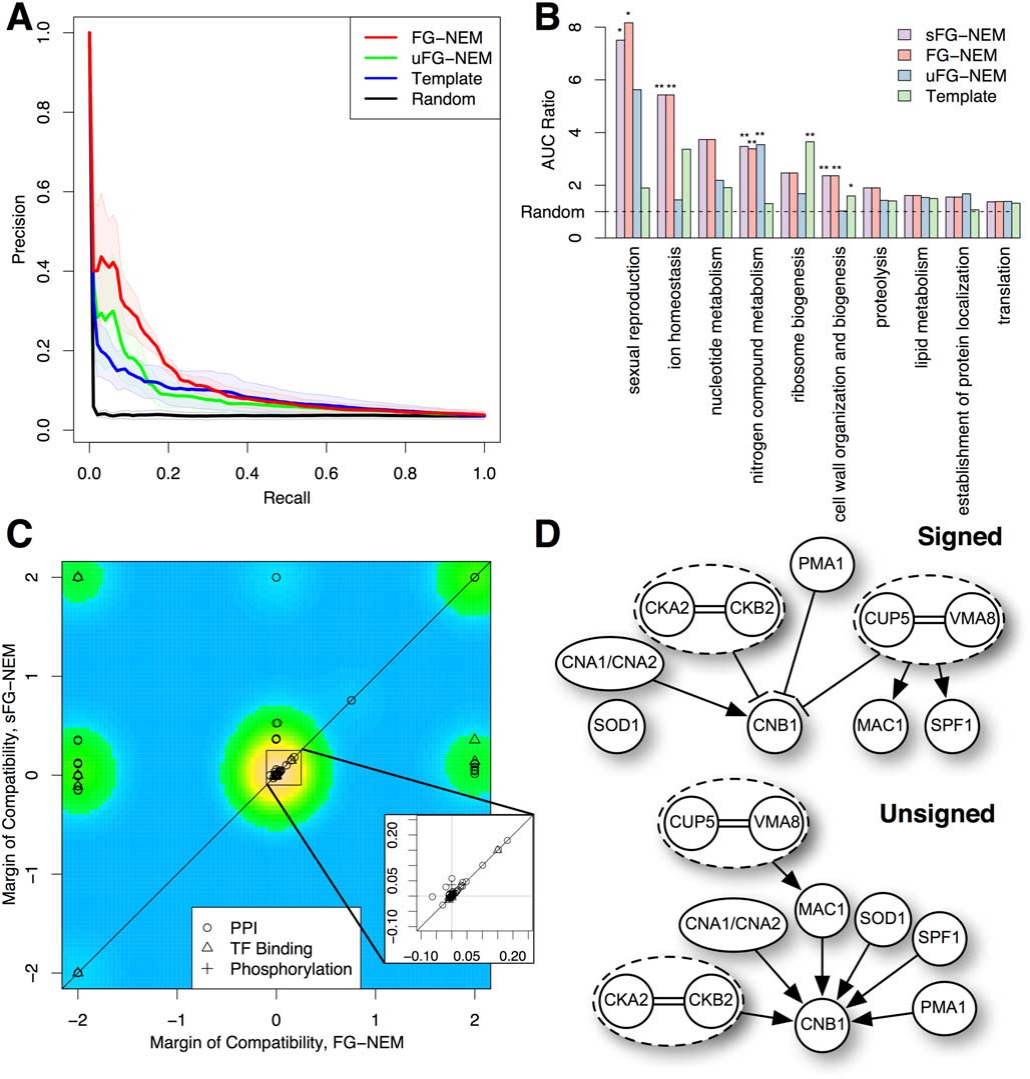
Yeast knock-out compendium predictions. (A) Precision/recall comparison. Each method's ability to expand a pathway was compared. Thick lines indicate mean precision and shaded regions represent standard error of mean calculated over the networks with the five highest AUCS from any of the tested methods. (B) Network expansion comparison. Networks were predicted for a non-redundant set of GO categories containing four or more S-genes in the Hughes et al. (2000) compendium and used to predict held-out genes from the same category (see Methods). The area under the curve (AUC) for each pathway was calculated for each method. AUC ratios (*y*-axis) were calculated for each method relative to the lowest AUC. (C) Compatibility of physical evidence and predicted S-gene interactions. Each point is the margin of compatibility (MOC, see Methods) of a predicted genetic interaction to high-throughput physical interaction data when physical interaction evidence was used (*y*-axis) and when it was not used (*x*-axis). Coloring indicates two-dimensional density estimation of points. Inset shows detail of the highest density region. Prediction methods that are significantly better than the lowest performing method, excluding random, at the 0.05 level (*) and 0.01 level (**) were determined by a proportions test on the top 30 predictions from each method. (D) Predicted S-gene networks for the ion homeostasis pathway. Shown are predicted networks from the FG-NEM method (Signed) and the uFG-NEM method (Unsigned). Arrows indicate activating interactions and tees indicate inhibiting interactions. The absence of a link between a pair of S-genes indicates the most likely mode for the pair was the non-interaction mode. Equivalence interactions are indicated with double lines and S-genes connected by equivalence are grouped into dashed ovals.

The accuracy of FG-NEMs for expanding each pathway to include new genes was measured. The likelihood of attachment ratio (LAR) score for each gene in the genome was calculated and the area under the precision-recall curve (AUC) was computed (see Methods). For each pathway, an AUC ratio was then calculated by dividing each method's AUC by the AUC calculated from randomly guessing E-genes for attachment to the network. Pathways sharing 25% or more of their genes with another pathway of higher AUC were ignored. Five non-redundant pathways were found that had AUCs significantly better than random guessing for at least one of the methods. While the precision of FG-NEM over uFG-NEM was not significant at any specific recall range, its overall higher precision across a broad range of recalls reflects a systematic improvement. Figure 4A shows the precision-recall curves averaged across these five pathways. The AUC ratios for the selected pathways are shown in Figure 4B and are sorted by the AUC achieved under the best-performing method.

Except for ribosome biogenesis, FG-NEMs performed comparably or better than uFG-NEMs and TM (Figure 4B and Table S1). For sexual reproduction, ion homeostasis, and cell wall, FG-NEM outperformed the other methods by the largest margins, outperforming TM by a ratio of 4.17, 3.98, and 2.64 respectively. The signaling networks of both sexual reproduction and ion homeostasis consist of several inhibitory interactions [35],[36], consistent with FG-NEM's ability to capture negative as well as positive regulatory interactions. TM may perform the best on ribosome biogenesis because the proteins involved in ribosome assembly are all tightly coregulated and their knock-outs lead to severe (and uninformative) effects. The signatures of expression changes for the ribosome biogenesis genes are not distinct from arbitrary genes because knocking out any of the ribosome biogenesis genes leads to drastic fitness defects in yeast and a concomitant alteration in gene expression to many genes in the genome.

Incorporating physical interaction priors showed little effect on network expansion performance. For most of the pathways, the performance of sFG-NEMs was indistinguishable from its unsupervised counterpart. A slight improvement was seen for the nitrogen metabolism pathway. Incorporation of structural priors adds activation from GLN3 to YEA4, and from ARG80 to ARG5,6, and slightly boosts the predictive power of the network. Thus, FG-NEM can usually identify new pathway genes in the unsupervised setting as well as when known interactions are provided.

Interestingly, the largest change in performance resulting from the use of prior information was a small drop observed for predicting genes involved in the sexual reproduction pathway. We investigated this decrease and found that using protein-DNA priors forced the placement of a transcription factor STE12 to the top of the pathway, whereas placement toward the bottom seemed to better fit the expression changes. Consequently, FG-NEM ranks the sexual reproduction E-genes higher than sFG-NEM.

On average, physical interaction priors increase the compatibility of FG-NEM predictions with high-throughput physical data. A leave-one-out analysis was used to test the ability of physical interaction data to improve pair-wise interaction predictions. To compare improvement in network structure prediction, we calculated the *margin of compatibility* (MOC) to reflect how well predicted interactions match held-out physical evidence (see Methods). Negative MOCs are assigned to predicted interactions that are incompatible with the physical evidence, while positive MOCs assigned to compatible predictions. For each held-out physical interaction, a network was computed using all other physical interaction data. Figure 4C shows the MOC of using priors plotted against the MOC without priors.

Of the 163 physical interactions, 104 (63%) have higher while 43 (26%) have lower MOC in sFG-NEM than FG-NEM. Of these 43, 33 have positive MOCs for both approaches (i.e. both agree with the physical evidence). Notably, of the 93 that achieved higher compatibilities in sFG-NEM, 38 (23%) became compatible only when the physical evidence was included. One example is the interaction between CDC42 and FAR1 in the sexual reproduction pathway. FAR1 acts downstream of CDC42 in the pheromone response signal cascade. The FAR1 gene deletion shows little expression change and is not placed downstream of CDC42 even though CDC42 is placed at the top of the signaling cascade by FG-NEM. With the inclusion of other structural priors, FAR1 is correctly placed downstream of CDC42. Thus, incorporating known interactions, even from possibly noisy high-throughput sources, can increase the likelihood of finding other interactions. However, the caveat is that such information may force a poorer fit to the observed expression data which could decrease the accuracy of frontier expansion.

#### Predicted inhibition in ion homeostasis pathway

FG-NEMs achieved significant improvement over the unsigned variant on the ion homeostasis pathway. To gain insights into the structural predictions underlying the difference in performance of the methods, we compared the predicted S-gene networks of the FG-NEM and uFG-NEM methods for this pathway (Figure 4D). In budding yeast, calcineurin regulates gene expression and ion transport in response to calcium signals by dephosphorylating the transcription factor Crz1p, thus allowing Crz1p to rapidly translocate from the cytosol to the nucleus [37]. Conversely, the casein kinase homolog Hrr25p binds to and phosphorylates Crz1p to functionally antagonize calcineurin signaling in yeast [38]. FG-NEMs predicted an ion homeostasis gene network that is comprised of a number of biologically relevant links where CNA1 stimulates CNB1, the casein kinase 2 subunit genes CKA2 and CKB2 are equivalent and repress CNB1, and the vacuolar proton pump subunits CUP5 and VMA8 are likewise equivalent and repress CNB1 (Figure 4D).

Both the FG-NEM and uFG-NEM correctly predicted the equivalence of CKA2 and CKB2 which together form a complex. Of the top fifteen frontier genes predicted by FG-NEM, eight are annotated by GO as involved in ion homeostasis (Table S2), FRE2 is involved in ion transport, YGL039W is an oxidoreductase, and ARO9 is involved in amino acid catabolism. In contrast, only one of the top uFG-NEM frontier genes, GRX4, is annotated by GO as involved in ion homeostasis. Examining the top 20 true positives predicted to be attached by FG-NEM, 19 were found to be predicted to be repressed by their S-gene. These true positives were not predicted to be attached to the network by uFG-NEM. Thus, the inability to make use of the explicit depression of E-genes may contribute to the poorer performance of the unsigned method.

### Application to Colon Cancer Invasiveness

We applied the FG-NEM approach to a human colon cancer invasiveness network elucidated by Irby et al. (2005) [26]. In this work, the authors identified several “tiers” of genes implicated in the invasion process under the control of SRC kinase. Genes were included in a tier if their knock-downs were found to produce a significant drop in the invasive potential of HT29 colon cancer cells as defined by invasion through Matrigel. To identify additional genes involved in the invasion process, the authors measured gene expression under an RNA interference knock-down of each gene in the tier. Genes whose expression was lower in the knock-downs producing loss-of-invasiveness, and higher in knock-downs that did not produce loss-of-invasiveness, were considered candidates for inclusion in the next tier. In this fashion, each tier was formed by knocking-down each candidate gene and assaying for loss-of-invasion in Matrigel.

#### Data

We applied FG-NEMs to the five S-genes from the second tier of Irby et al. (2005). These five human genes are cytokeratin 20 (KRT20), transcription factor Dp-1 (TFDP1), DEAH (Asp-Glu-Ala-His) box polypeptide 32 (DHX32), ribosomal protein L32 (RPL32), and glutaminase (GLS). Knock-down of each second-tier S-gene has been demonstrated to significantly reduce the invasion phenotype of HT29 colon cancer cells (Irby et al., 2005). KRT20 has historically served as a diagnostic marker for colorectal carcinoma [39], whereas high expression of ribosomal protein L32, glutaminase, and DEAD/H box polypeptides has been associated with various cancers and metastatic lesions [40],[41]. For this study, S-genes from the first tier were excluded as the expression profiles from the knock-down experiments were collected on a different microarray platform and therefore cross-platform normalization issues could potentially impact the results. The Expression Factor parameters were estimated from genes found to be up- or down-regulated by running the Statistical Analysis of Microarrays algorithm (SAM) [5], with a False Discovery Rate of 1%, on gene expression data collected on a panel of knock-downs. Using the differentially expressed genes yielded an estimate of 1.75 for the mean log_2_ ratio of the inhibited E-gene distribution (−1.75 for the activated E-gene distribution), and a standard deviation of 0.5 for the Gaussian mixture model (see Methods). Several of these knock-downs led to loss-of-invasiveness while others produced invasive growth in the Matrigel assay as reported by Irby et al. (2005). The hybridization data and associated normalization information can be accessed from the Gene Expression Omnibus (GEO) database [42] under the series accession number GSE11848 and associated platform accession number GPL6978. A subset of this data containing the SAM-selected E-genes can be obtained from Dataset S1.

#### Cancer invasion network identification

**Figure 5:**
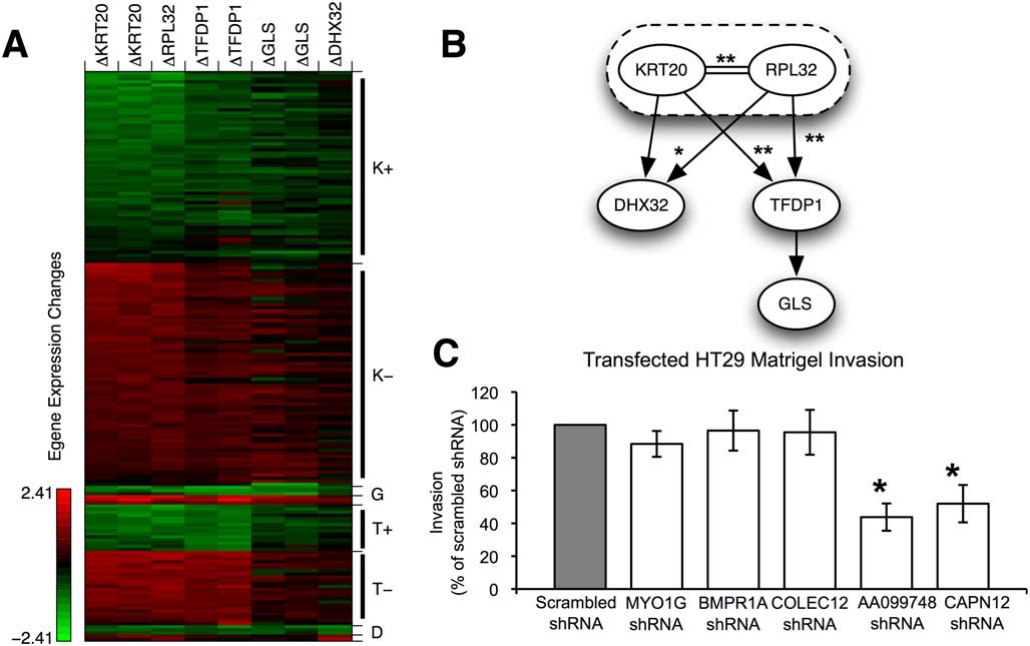
Invasive colon cancer network predictions. (A) Expression changes of selected E-genes following targeted S-gene knock-downs in HT29 colon cancer cells. Gene expression was measured in HT29 cells treated with a shRNA specifically targeting an S-gene (column of the matrix) relative to cells treated with a scrambled control shRNA (Irby et al., 2005). Colors indicate putatively inhibited E-genes (rows of the matrix) with up-regulated levels relative to control (red), activated E-genes with down-regulated levels relative to control (green), and unaffected E-genes with expression levels not significantly different from control (black). Biological replicates were available for KRT20, TFDP1, and GLS knock-downs. Genes were sorted by their attachment point and then by their LAR scores. (B) Cancer invasion network predicted by FG-NEM. For each pair of S-genes, the most likely interaction mode is shown. The same conventions used for illustrating interactions predicted for the yeast networks were used here. Some interactions were found to be significant at the 0.05 level (*) or 0.01 level (**) using a permutation test (see Methods). KRT20 and RPL32 were predicted to be equivalent and are therefore grouped together in a dashed oval. (C) Matrigel invasion assay in HT29 colon cancer cells. Genes predicted to be significantly attached to the network, CAPN12 and expressed sequence tag AA099748, resulted in a loss of the invasiveness phenotype when knocked-down by RNA interference. Genes not significantly attached to the network, MYO1G, BMPR1A, and COLEC12, did not result in significant loss of the invasive phenotype. A scrambled non-sense sequence also served as a negative control and did not result in a loss of HT29 cell invasiveness. Gene knock-downs in HT29 cells were validated by quantitative real time RT-PCR where mRNA levels of targeted genes were decreased by 70–80% compared to scrambled control shRNA-treated cells (data not shown). Data shown are the mean±S.E. of five independent experiments performed in quadruplicate. *Significantly different from scrambled control shRNA-treated cells (P<0.05) by ANOVA and post hoc Tukey test.

We applied FG-NEMs to recover a network for the second-tier genes. We included E-genes that demonstrate a robust and significant effect under at least two of the knock-downs included in the Irby et al. (2005) study. We selected genes whose log_2_ ratios differ by less than 0.5 in replicate arrays and had an absolute log_2_ expression change at least equal to the mean absolute level of the activated distribution (1.75) in at least two arrays. Using these criteria, we identified 185 E-genes to use for model inference. Figure 5A shows the expression data of these E-genes plotted in order of their predicted attachment points as identified by FG-NEMs. For the most part, E-gene expression changes moved in the same direction following knock-down across the panel of five S-genes, indicating the presence of mostly stimulatory links among the S-genes (Figure 5A). This is in contrast to Figure 1A, where expression changes of a single E-gene move in the opposite direction following knock-down of S-genes connected by an inhibitory link. The absence of inhibitory links among S-genes is expected since, according to the selection criteria, all of the S-genes were found previously to act in the same direction (invasion promotion). The method does find many inhibitory links to E-genes, which dramatically increases the fit of the model on the data points. These predicted attachment signs provide information about how an E-gene's involvement in the invasion process can be tested in follow-up experiments. The model predicts that invasion can be suppressed by knocking down genes connected by stimulatory attachments or by over-expressing genes connected by inhibitory attachments.

FG-NEM recovered the network shown in Figure 5B. KRT20 and RPL32 are predicted to be equivalent. Also, the model predicts TFDP1 and DHX32 are downstream of KRT20 and RPL32. The equivalent interaction of KRT20 and RPL32 received significantly high likelihoods (*P*<0.001) as well as a strong excitatory downstream connection to TFDP1 (*P*<0.001). There is a significant excitatory connection between KRT20/RPL32 and DHX32 based on one series of knock-down experiments specifically targeting KRT20 (*P* = 0.006), although a second knock-down experiment (using a silencing RNA differing from the first series that targets a different region of the KRT20 mRNA) resulted in a weaker connection (*P* = 0.534). Consequently, one could designate this link as deserving of follow-up functional studies (e.g. promoter analysis or chromatin immunoprecipitation). Though GLS is connected to the network, the likelihood of interaction was not strong enough to be significant (Figure 5B). Hence, the GLS connection may require future knock-downs of additional S-genes coupled with gene expression profiling in order to resolve its tentative connection.

The FG-NEM model predicts that TFDP1 is at the bottom of the signaling cascade, which may reflect its role as part of the E2F transcriptional complex in targeting the expression of downstream genes that promote cell proliferation and invasion [43],[44]. The ribosomal subunit, RPL32 is curiously placed upstream of the DP1 transcription factor and at an equivalent level with the structural molecule KRT20. Aberrant expression of ribosomal proteins has been noted in a variety of cancers, although the molecular consequence of these expression changes is unknown [45]. It has been postulated that ribosomal proteins may play an important extraribosomal role (i.e. beyond translation) in the oncogenic transformation process [45].

Because the number of S-genes in the second tier is small, we compared the heuristic pair-wise search employed by FG-NEM to an exhaustive model search. If the heuristic approach is reasonable, it should identify network models that are among the highest scoring models identified by brute-force enumeration. To perform a brute-force search, we generated 1000 random networks among the five second-tier genes. For each network, we calculated the data likelihood using message passing. Out of the 1000 randomly enumerated networks, the recovered network for the second-tier genes had a likelihood higher than 997 of the random networks. Interestingly, all three of the random networks with higher scores had identical structures to the network recovered by FG-NEM except that all three networks differed in their attachment of DHX32 and GLS. This result demonstrates that the pair-wise heuristic search employed by FG-NEM successfully identifies high-scoring networks in the space of all networks. While we need to test the trend for increasing network sizes, these results are promising for scaling up to larger networks in which exhaustive search will not be feasible.

#### Cancer invasion frontier expansion

We used the highest-scoring model recovered by the FG-NEM to search for additional genes involved in colon cancer invasiveness by sorting each gene by its LAR score (see Methods). We found 19 positive and 31 negative attachments with significant probabilities (Table 1 and Table S3). Significance of the attachments was assessed by permuting each E-gene's observations, relearning a FG-NEM network, and computing its LAR score to construct an empirical null distribution of LARs. The E-genes with the highest attachment probabilities and positive LAR scores found to be significant via permutation testing are shown in Table 1.

**Table 1:** Top frontier genes for colon cancer invasiveness ranked by LAR score (see Methods) and filtered for significance as determined by data permutation test (see Methods).

LAR^a^	E-Gene	S-Gene	E-Gene Description
18.79	CHORDC1^b^	GLS	Cysteine and histidine-rich domain-containing 1
11.35	RNF32	GLS	Ring finger protein 32
10.93	TSP50	TFDP1	Testes-specific protease 50
10.02	HS3ST1^d^	KRT20	Heparan sulfate (glucosamine) 3-O-Sulfotransferase 1
6.85	CHMP4C^c^	TFDP1	Chromatin modifying protein 4C
6.76	ADAM19^b^	KRT20	ADAM metallopeptidase domain 19 (meltrin beta)
6.34	CYP3A43	KRT20	Cytochrome P450, family 3, subfamily A, Polypeptide 43
5.97	SPTLC3^b^	TFDP1	Serine palmitoyltransferase, long chain base subunit 3
5.25	PLEKHM3^b^	KRT20	Pleckstrin domain containing
4.92	KRT13	TFDP1	Keratin 13
4.28	CAPN12	KRT20	Calpain 12
3.87	C1orf34^b^	KRT20	Hypothetical
3.54	ZNF350	KRT20	Zinc finger protein 350
3.53	ADAM9	TFDP1	ADAM metallopeptidase domain 9
2.75	SLC2A1^b^	KRT20	Solute carrier family 2
2.38	TCTEX1D1	TFDP1	Tctex1 domain containing 1
2.23	STK24	KRT20	Serine/threonine kinase 24
2.05	DDX58	KRT20	DEAD (Asp-Glu-Ala-Asp) box polypeptide 58
2.01	GFAP	KRT20	Glial fibrillary acidic protein

a Natural logarithm of likelihood of attachment score (see Methods).

b EST is inside an intron of this gene.

c EST is on the 3′ end of this gene.

d EST is on the 5′ end of this gene.

Many of the genes in Table 1 have roles consistent with cancer cell invasion. For example, three E-genes encode proteases, including the metalloproteases ADAM9 and ADAM19. The metalloproteases represent a class of transmembrane proteins that are known facilitators of cell migration and invasion by proteolytic cleavage of extracellular matrix components [46]. Interestingly, ADAM21 is included among the first tier genes of Irby et al. (2005). This demonstrates that FG-NEM is able to identify two additional family members of this first tier gene even though it was not included in the S-gene set used in network learning. Glial fibrillary acid protein (GFAP) and Testes-specific protease 50 (TSP50) are also included in Table 1. GFAP is known to interact with the oncogenic tyrosine kinase SRC [47] and involved in astrocyte tumor invasiveness [48], while TSP50 has been shown to be differentially regulated in both breast and testicular cancer [49],[50]. Thus, FG-NEMs predict that an expanded set of proteases may play a role in the colon cancer invasion process. Also included among the set of genes in our expanded invasion network is a second keratin family member, keratin 13 (KRT13), which is consistent with the previous identification of KRT20 in the second tier and may reflect a structural underpinning needed for invasion. Several of the genes in Table 1 represent novel connections of genes to the colon cancer invasiveness pathway. For example STK24, is a highly conserved protein whose homolog in *S. cerevisiae*, STE20, is involved in signal transduction of pseudo-hyphal growth [51]. It is intriguing to consider the possibility that part of the invasiveness pathway could be due in part to the aberrant regulation of an ancient cell migration process that dates back to single-cellular organisms.

The E-genes with positive LAR scores constitute the network “frontier” of the cancer invasiveness pathway in that they are predicted to directly interact with the second-tier genes. From among the 38 genes with positive and significant LAR scores, two were arbitrary selected to test for a loss-of-invasiveness phenotype in HT29 cells as defined by invasion in Matrigel. We selected CAPN12 and expressed sequence tag AA099748 from Table 1 for gene knock-down experiments. CAPN12 is a member of the calpain gene family, which has been shown to have fibrillin activity. Genbank EST accession AA099748 aligns to the genome 3′ to the gene CHMP4C, along with the EST AW440175, both from cancer tissues. Additionally, the amino acid translations of these ESTs align to the N-terminus of CHMP4C with 48% identity. The C-terminal tail of CHMP4C was recently shown [52] to be bound by the apoptosis inhibitor PDCD6IP, suggesting that the cancer-specific splice form of CHMP4C may have altered binding behavior with PDC6IP. PDC6IP also has been implicated in a broad array of membrane associated processes, including cell adhesion [53]. Serving as negative controls, we performed knock-down experiments for three E-genes that had low attachment probabilities, namely MYO1G, BMPRIA and COLEC12. As correctly predicted by FG-NEM, both E-genes with high LAR scores produced significant loss of invasion while all three E-genes with low LAR scores did not lead to loss-of-invasion in the Matrigel assay (Figure 5C).

## Discussion

The factor graph nested effects model (FG-NEM) provides a general methodology for inferring networks from knock-down phenotypes. Our results extend the nested effects models in three significant ways: 1) we provide a means for efficiently searching for large S-gene networks using inference on a factor graph that can also incorporate prior information; 2) our method distinguishes activating from inhibiting interactions; and 3) we show that NEM attachment can be used successfully to expand the network to new pathway members. Our results on simulated and yeast networks suggest explicitly modeling inhibition and activation, rather than treating as generic interactions or effects, leads to higher accuracies for recovering known interaction networks and identifying members of the a pathway.

Applying FG-NEM predictions to a series of follow-up experiments in an HT29 colon cancer cell line model has identified new gene members of the tumor invasiveness pathway. Specifically, shRNA-mediated knock-down of two genes predicted to be connected to the original rudimentary network of Irby et al. [22] led to a significant loss of invasiveness whereas three genes predicted not to be connected did not result in a loss of invasive phenotype following knock-down. Our results suggest FG-NEM improves upon the iterative strategy followed by Irby et al. [26]. The iterative procedure of Irby et al. produces a graph in which genes in a tier are connected only to genes in the next tier. The graph does not necessarily reflect the signaling events underlying invasion. Rather, it encodes the chronological order by which the genes were elucidated. In contrast, FG-NEM seeks a structured model that relates the genes within and across tiers, which may provide a better understanding of the signaling and regulatory events leading to cancer cell invasion. In addition, rather than using differential expression as a criterion to expand the network, FG-NEMs search for genes that have expression changes coherent with the dependencies encoded in the learned structure. FG-NEMs were able to identify two confident relationships among the genes in the second tier that the previous iterative strategy of Irby et al. (2005) could not identify. The equivalence of RPL32 and KRT20 as well as the downstream relation of TFDP1 and DHX32 to these two genes is a first step toward refining the architecture of the colon cancer invasiveness network. Moreover, these findings suggest that RPL32 may play an important extraribosomal function by regulating TFDP1 mRNA expression.

We envision applying the FG-NEM approach within an iterative computational-experimental framework. As a network is expanded, the frontier genes of one round of investigation can be included as S-genes in subsequent rounds. Iteration will therefore provide larger sets of S-genes on which to infer networks. While the primary data used for such network expansion is based on gene expression data, it will be intriguing to investigate whether a variety of transcriptional and non-transcriptional interactions can be recovered with this approach. There are many examples of coupling between transcription and non-transcriptional interactions in biological systems. An E-gene *e* attached to S-gene *A* does not necessarily imply the signaling between *A* and *e* is transcriptional in nature. Consider a metabolic cascade in which *A*'s product produces substrate *s_1_*, which is converted to *s_2_* by *e*, which is a substrate of an enzyme encoded by a second S-gene *B*: *A*→*s_1_*→*e*→*s_2_*→*B*. Furthermore, assume that the cell has a mechanism to “sense” the amount of *s_1_* and that this mechanism controls the transcription of *e*. Deletion of *A* in this scenario will lead to a decrease in *s_1_* which will cause *e*'s expression to decrease. Thus, promotion of *e* to the network in this case could reveal a new gene involved in “signaling” via metabolic transformation. The *lac* operon in bacteria uses a similar coupling between the expression of the enzymes in the pathway to sense cellular concentrations of lactose [54]. As another example, consider the metazoan phosphorylation cascade in which signaling between S-genes is coupled to their own mRNA production. Phosphorylation of the transcription factor heterodimer Jun and Atf2 by Jnk then promotes transcription of the JUN gene [55]. More Jun protein is made, leading to dimerization with another protein, Fos, which activates transcription of other downstream genes. Knock-down of JNK results in transcriptional down-regulation of JUN. Thus, promotion of JUN from an E-gene to the network would reveal a member of the pathway involved in post-translational signaling even though it was detected through transcriptional perturbation.

Several aspects of the method could be improved upon in the future. The method could be extended to use over-expression of S-genes in addition to knock-downs. Over-expression of an S-gene would be expected to have an opposite effect on downstream E-genes compared to the E-gene effects observed under the S-gene's knock-down. Thus, the E-gene responses could be compared to an expanded list of interaction modes, derived by flipping the scatter-plots in Figure 1 by either the *x*-axis, *y*-axis, or both axes depending on if S-genes *A*, *B*, or both are over-expressed.

In this study of the colon cancer invasiveness pathway, S-gene interaction configurations were forced to reflect transitive connections but did not incorporate any external biological information. Additional knowledge, such as gene coexpression groups, or protein-protein interaction potentials, could be incorporated into the prior for making inferences about the cancer invasiveness pathway. For example, several gene expression experiments on invasive colon cancer cell lines are available in GEO [42]. It would be interesting to extract sets of genes that are up- or down-regulated in invasive versus non-invasive cancer cells consistently across multiple studies. Any S-genes present in such recurrent sets could be associated with higher pair-wise interaction priors than arbitrary S-gene pairs. However, since we observed a decrease in performance for pathway expansion on the yeast networks, we chose not to attempt this at this time.

We modeled transitivity using deterministic factors. While this provides an intuitive interpretation of such constraints and increases the speed of convergence of message passing, relaxing these constraints to general belief potentials could allow a broader exploration of the search space. Imposing transitivity in the current framework disallows cycles of inhibitory links. However, it is possible to extend our method to incorporate such cycles, in which new interaction modes are introduced. For example, the cycle A→B⊣C→A would imply B⊣A, which could be modeled using a new type of interaction mode capturing A's activation on B and B's inhibition on A.

The methods could be extended to incorporate richer information such as degrading signals and higher-order knock-downs (single, double, triple, etc) as in Carter et al. (2007) [14]. Our formulation assumes that the effects of a knock-down do not degrade along a pathway and also neglects combinatorial interactions of multiple genes. FG-NEMs allow higher-order knock-down combinations to be incorporated into a search for high-scoring networks. Using only single knock-downs, it may be impossible to identify certain relationships such as the synthetic effects of two parallel pathways converging to one gene. In principle, FG-NEM can handle higher-order relations by extending the pair-wise likelihood term to contain three or more genes. However, the large numbers of possible combinatorial relations and combinations of knock-down experiments required to elucidate the relations, as well as the propagation of complexity along the pathways, would make the problem more difficult.

In our network expansion approach, we assumed genes whose expression levels are well-explained by the model are of more interest for subsequent rounds of experimentation, although there are other ways to approach this question from an experimental design perspective. For example, it would be conceivable to test whether selecting genes based on reducing a measure of uncertainty across models leads to better gene selection as in [13]. An “active learning” approach prioritizes knock-down experiments based on the reduction of expected entropy of high-scoring models. The “informative” experiments would effectively disambiguate the models which explain the existing data. Fewer experiments might then be needed to narrow down a unique model of the underlying system [56],[57].

Finally, the approach could be applied to the unsupervised discovery of regulatory interactions among E-genes rather than S-genes. In recent work, Sahoo et al. (2008) [58] applied a pair-wise scoring approach for detecting Boolean implications based on gene expression changes observed across hundreds of microarray studies. Similarly, FG-NEMs could use the expression changes measured across a diverse array of conditions to score gene pairs against interaction mode templates (Figure 1C) to determine if a specific regulatory interaction is more probable than non-interaction.

### Conclusions

We applied FG-NEMs to discover a human signaling network among genes involved in colon cancer cell invasiveness. The method formalizes and extends analysis of genetic interactions using high-dimensional quantitative phenotype data in the form of gene expression changes observed under specific perturbations. It makes explicit use of the knock-downs of known members of a pathway to identify how the members interact with one another and for identifying new members. The method predicts several genes with new roles in the cancer invasiveness process, two of which were verified to act in the pathway based on an *ex vivo* invasion assay. Thus, the FG-NEM approach may be a powerful tool for inferring regulatory connections and for identifying new partners of genes known to operate in a process of interest. The application of structured causal models for pathway identification and expansion promises to greatly accelerate the discovery of genetic pathways from genetic knock-downs and other intervention-based experiments.

## Supporting Information

Dataset S1 Colon cancer invasion data for SAM-selected E-genes. Sheet 1. Selected E-genes and their expression. Sheet 2. Input to SAM for determining parameters of the Gaussian mixture in the Expression Factors. Sheet 3. SAM results used for determining the parameters of the Gaussian mixtures in the Expression Factors.
(6.09 MB XLS)

Figure S1 Observed inhibitory effects and signaling in yeast compendiums Evidence for inhibition from measured responses of knockdown, and from annotation in curated pathways.
(0.09 MB PDF)

Figure S2 Comparison of uFG-NEM and exhaustive NEM model search for structure recovery.
(0.07 MB PDF)

Figure S3 Estimating difference in gene expression between activation and inhibition.
(0.08 MB PDF)

Figure S4 Accuracy of network recovery as a function of S-gene knowledge and number of microarray replicates, and E-gene inhibition.
(0.10 MB PDF)

Table S1 Yeast Knockout Compendium Pathway AUC. AUC and AUC-ratios for expansion of Yeast pathways.
(0.03 MB XLS)

Table S2 Ion Homeostasis Frontier Genes. Genes most likely to be attached to the ion homeostasis network for both the FG-NEM and uFG-NEM methods. Genes are sorted by LAR.
(2.75 MB XLS)

Table S3 Invasiveness E-gene LAR Scores. Connection point, connection strength, and connection significance of E-genes in colon cancer network.
(0.10 MB XLS)

Text S1 Supplemental methods and results.
(0.12 MB DOC)
