# Supplementary material for: A Factor Graph Nested Effects Model To Identify Networks from Genetic Perturbations
Source: PLoS Comput Biol. 2009 Jan 30;5(1):e1000274. doi: 10.1371/journal.pcbi.1000274 (PMC2613752; doi:10.1371/journal.pcbi.1000274)
Supplement: Figure S1 — Observed inhibitory effects and signaling in yeast compendiums Evidence for inhibition from measured responses of knockdown, and from annotation in curated pathways. (0.09 MB PDF) [file pcbi.1000274.s002.pdf]

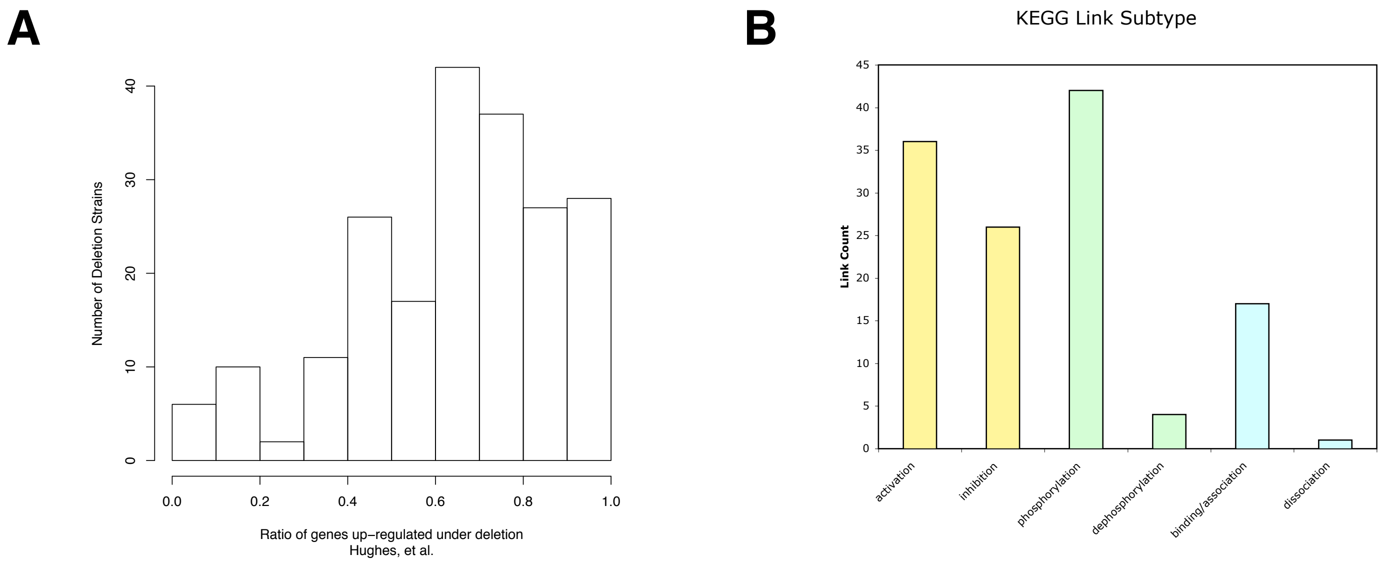

Figure S1. Observed inhibitory effects and signaling in a yeast compendium. A. Histogram of percentage of up-regulated gene expression from gene knockdown in the Hughes *et al.* (2000) compendium. In each deletion strain, gene expression changes with a p-value better than 0.05 were selected, and then assigned to up-regulated or down-regulated according to their expression log-ratio. The percentage of each strain's up-regulated expression change is plotted in the histogram. Presence of up-regulated expression under gene deletion is evidence of an inhibitory interaction close to the deleted gene. **B. Histogram of interaction types in *Saccharomyces cerevisiae* in the KEGG pathway database.** In the KEGG ontology, each interaction may be categorized with more than one label. Activation and inhibition are antonyms, as are expression and repression.
