# Supplementary material for: A Factor Graph Nested Effects Model To Identify Networks from Genetic Perturbations
Source: PLoS Comput Biol. 2009 Jan 30;5(1):e1000274. doi: 10.1371/journal.pcbi.1000274 (PMC2613752; doi:10.1371/journal.pcbi.1000274)
Supplement: Figure S2 — Comparison of uFG-NEM and exhaustive NEM model search for structure recovery. (0.07 MB PDF) [file pcbi.1000274.s003.pdf]

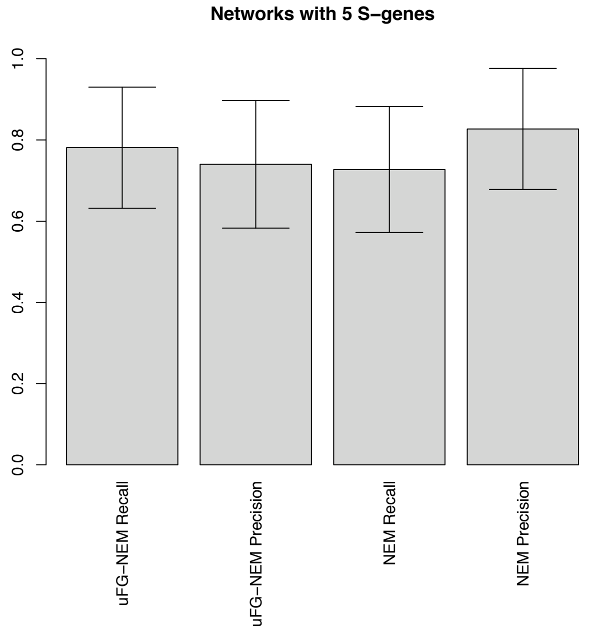

Figure S2. Comparison of uFG-NEM and exhaustive NEM model search for structure recovery. Each bar shows the mean and standard deviation of precision or recall for S-gene links over 100 artificial networks with five S-genes. Networks were generated with no inhibition and an average of 20 E-genes per S-gene. Expression was generated for one replicate using standard deviation of 1 and a mean for the down distribution of -1.75. Precision and recall for both uFG-NEM and exhaustive NEM search do not differ significantly.
