# Supplementary material for: A Factor Graph Nested Effects Model To Identify Networks from Genetic Perturbations
Source: PLoS Comput Biol. 2009 Jan 30;5(1):e1000274. doi: 10.1371/journal.pcbi.1000274 (PMC2613752; doi:10.1371/journal.pcbi.1000274)
Supplement: Figure S3 — Estimating difference in gene expression between activation and inhibition. (0.08 MB PDF) [file pcbi.1000274.s004.pdf]

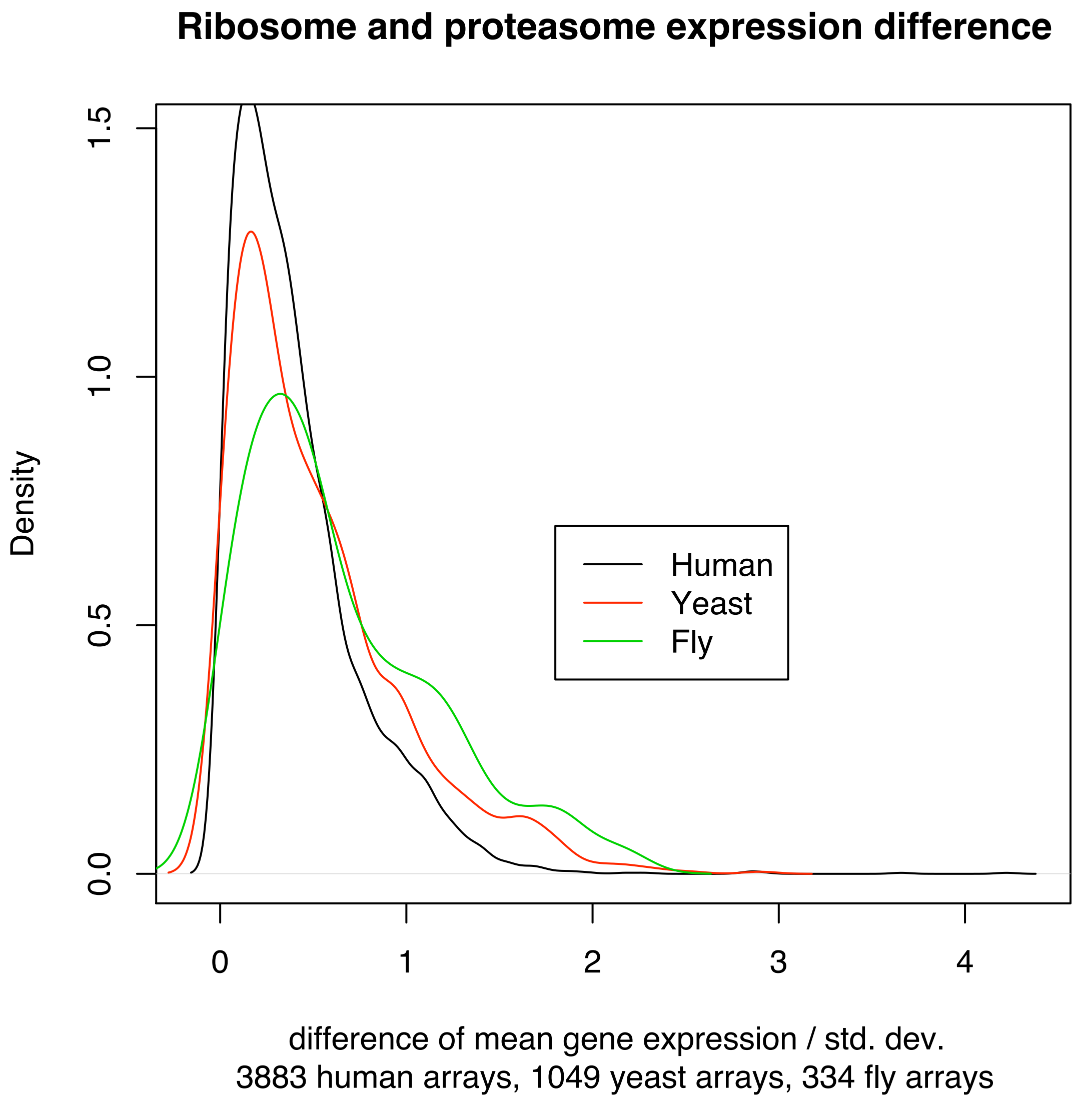

Figure S3. Estimating the difference between up- and down- distributions using proteasome- and ribosome- related genes as contrast sets. In *C. elegans, S. cerevisae,* and *H. sapiens,* we determined members of the ribosome by collecting all genes with a GO annotation term that begins with "ribosome." Similarly, we constructed a proteasome gene set in each species from any gene annotations with a GO term beginning with "proteasome." We collected two-channel microarrays from databases and supplementary material for a total of 334 *C. elegans,* 1049 *S. cerevisiae,* and 3883 *H. sapiens* microarrays. Within each microarray we calculated the mean and standard deviation of the set of ribosome genes and the set of proteasome genes. For each microarray we then calculated the absolute value of the difference of the mean of ribosome and proteasome expression, and divided by the geometric means of the standard deviations. Finally, we plotted density estimates for each species.
