# Supplementary material for: A Factor Graph Nested Effects Model To Identify Networks from Genetic Perturbations
Source: PLoS Comput Biol. 2009 Jan 30;5(1):e1000274. doi: 10.1371/journal.pcbi.1000274 (PMC2613752; doi:10.1371/journal.pcbi.1000274)
Supplement: Figure S4 — Accuracy of network recovery as a function of S-gene knowledge and number of microarray replicates, and E-gene inhibition. (0.10 MB PDF) [file pcbi.1000274.s005.pdf]

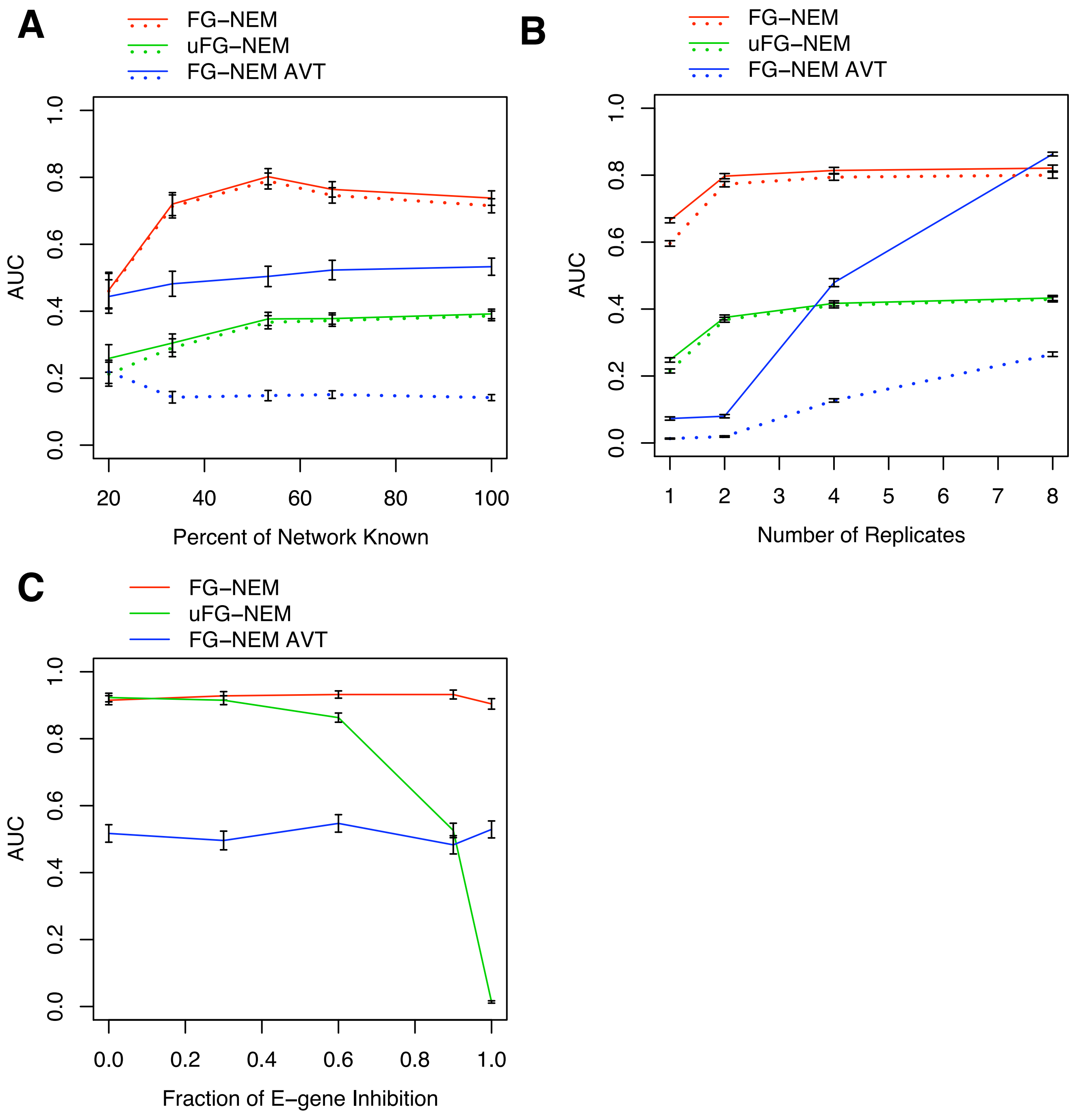

Figure S4. Accuracy of network recovery as a function of S-gene knowledge and number of microarray replicates, and E-gene inhibition. A. Influence of incomplete pathway knowledge on network recovery. Expression data for a subset of S-genes was made available for network recovery. AUC (*y*-axis) plotted as a function of the percent of S-genes having available data (*x*-axis). 40% inhibition and four replicates were used. Solid lines indicate AUC of S-graph connectivity. Dashed lines indicate AUC using both S-graph connectivity and sign. **B. Influence of replicates in network recovery.** AUC (*y*-axis) plotted as a function of varying numbers of microarray hybridization replicates (*x*-axis). Artificial networks contained 40% inhibitory interactions. **C. Influence of inhibitory E-gene connections in network recovery.** Networks with only activating S-gene interactions were reconstructed with varying amount of E-gene inhibition. At each inhibition level, 100 networks were used.
