# Supplementary material for: A Factor Graph Nested Effects Model To Identify Networks from Genetic Perturbations
Source: PLoS Comput Biol. 2009 Jan 30;5(1):e1000274. doi: 10.1371/journal.pcbi.1000274 (PMC2613752; doi:10.1371/journal.pcbi.1000274)
Supplement: Text S1 — Supplemental methods and results. (0.12 MB DOC) [file pcbi.1000274.s009.doc]

**Supplemental Results**

**Results on Artificial Networks**

**Influence of Microarray Replication on Network Recovery.** Uncertainty in microarray measurements is lessened by repeated hybridization either using biological or technical replicates. However, replication is costly or not possible in many situations. Because the datasets analyzed in this paper have at most two replicates, we were interested in testing the methods’ abilities to recover networks from data containing few replicates. We compared the performance of FG-NEM and uFG-NEM for varying numbers of replicates (Figure S4A). Our results are consistent with Markowetz *et al.*’s findings that the unsigned approach has a positive predictive value (PPV) about 0.1-0.15 higher when comparing four replicates to a single replicate [1]. In contrast, the new FG-NEM performs well even if a single hybridization is available for each S-gene knock-down. With one to four replicates, the performance of FG-NEM was significantly higher than uFG-NEM using either the original or the AVT data. As the number of replicates increased to eight, the two methods achieved comparable performance for uFG-NEMs run on the AVT data. To match the typical number of replicates included in microarray studies, four replicates were used for the rest of the artificial network experiments. At this number of replicates, FG-NEM significantly outperforms both uFG-NEM and FG-NEM AVT variants.

**Influence of the Extent of Incomplete Knowledge on Network Recovery**. In practice, we expect studies of well-characterized pathways to include many of the S-genes while studies of poorly characterized pathways will lack many of the pathway’s S-genes. A full network of fifteen genes was generated and then a subset of (*k*100)*% of the pathway genes was randomly selected from it for use as the set of S-genes. We tested the ability of both FG-NEMs and uFG-NEMs to recover the structure of the hidden network from S-gene subsets of different sizes by varying *k*.

As expected, the performance of both methods increased as the proportion of known S-genes was increased (Figure S4B). Methods achieve their best performance when at least half of the S-genes are known. This is promising as we can expect the best possible performance of the methods even if we use only half of the known underlying network. Conversely, all methods’ performances were low when smaller proportions of known S-genes were used, especially at 20-33% (3-5 S-genes out of fifteen). Compared to more complete S-gene subsets, using fewer S-genes introduces longer expected distances between any two S-genes, forcing the models to infer a greater proportion of indirect interactions. In the simulated networks, longer paths between S-genes will be associated with weaker signals compared to shorter paths. Therefore, the expression changes have more chances to diverge from an idealized nesting relationship compared to shorter paths. The FG-NEM remains more accurate than the unsigned counterpart for known levels of 33% and higher. For example, when 33% of the network is known, the FG-NEM method achieves an average AUC of 0.72, which is 50% higher than the AUC achieved by its unsigned counterpart.

**Supplemental Methods**

**Gaussians for E-gene Expression Factors**

The *P(X_eAr_ | Y_eA_)* terms give the probability of observed expression changes and are modeled with a mixture of Gaussians whose parameters are determined by an E-gene’s hidden state. A separate mean was estimated for each component distribution while the standard deviation was shared across the three component distributions. We assign a mean of zero to the background distribution *P(X_eAr_ | Y_eA_=0)* and means *μ* and –*μ to* the inhibited- and activated-distributions, *P(X_eA_ | Y_eA_=-1)* and *P(X_eA_ | Y_eB_=+1)*, respectively. The parameters σ and *μ-* were fit to each individual dataset as described in Results. Because the original implementation of NEMs was not efficient enough to apply to our datasets, we implemented an “unsigned” version of our approach by removing the inhibited-distribution. We refer to this version as the unsigned FG-NEM (uFG-NEM). Before using uFG-NEMs for use on large networks, we compared uFG-NEM to the original NEM approach and found they gave comparable results on small networks (See Figure S2).

**Transitivity Constraints**

For each interaction *φ_AB_*, let *f(φ_AB_)* denote the forward signal sign of *A* to *B*, and let *b(φ_AB_)* denote the back signal sign of *B* to *A*:

|  | 1 if *φ* ∈ {→,↔}  0 if *φ* ∈ {←,≠}  -1 if*φ* ∈ {─┤} |  | 1 if *φ* ∈ {←,↔}  0 if *φ* ∈ {→,≠}  -1 if*φ* ∈ {├─} |
| --- | --- | --- | --- |

The value of a transitivity constraint *τ_ABC_(**_AB_, $f$_BC_, $f$_AC_)* is then defined using Iverson notation as, where the expression [*predicate*] has value 1 if *predicate* is true, and 0 if false.

**Message Passing**

Each message µ(*φ_AB_*  τ*_ABC_*) or µ(τ*_ABC_ φ_AB_*), (denoted by µ and µ’ in Figure 2), respectively, consists of a length six vector, representing the source’s log-space valuation of six possible interaction types for *φ_ij_.* The message passing schedule consists of two-phase iterations. The first phase of an iteration calculates every message from an interaction variable to a transitivity constraint, µ(*I_ij_*  τ*_ijk_*). The second phase of an iteration calculates all messages from transitivity constraints to interaction variables, µ(τ*_ijk_ φ_ij_*). In order to calculate the first phase of the first iteration, every µ(τ*_ijk_ φ_ij_*) is set to [0,0,0,0,0,0]. To prevent non-convergence from cyclic activities, each new message is dampened by arithmetically averaging it with the message from the previous iteration as well as by normalizing each factor to variable message to sum to one. At the end of each iteration, the change in µ(τ*_ijk_*  *φ_ij_*) messages is calculated by forming a matrix of all messages, and calculating the Frobenius norm of the new message matrix and the previous message matrix. Message passing is terminated when the Frobenius norm is less than 0.01, or after 50 iterations.

During message passing the summation over *Y_eA_* is approximated with a maximization step. Max-sum approximation of a marginalization step has been shown to produce accurate results and converge efficiently for several factor graph applications to large problems including SAT and clustering [2,3]. The maximization chooses the highest probability configuration of attaching an effect at some point relative to a pair of S‑genes as well as the sign of the attachment.

**Physical Structure Priors**

Three classes of physical data were downloaded for use as interaction priors: protein-DNA interactions, phosphorylation target data, and protein-protein interactions (PPI). Protein-DNA interactions with a p-value less than 0.001 were selected from the study of Lee, *et al*. [4]. Kinase target data was taken from the study of Ptacek, *et al*. [5]. PPI data was downloaded from the BioGRID [6] on July 30, 2008. For each Gene Ontology (GO) [7] category under study, we selected any interaction between S-genes in that category, resulting in 27 Protein-DNA interactions, 4 phosphorylation interactions, and 64 PPIs for the GO sets discussed in this paper.

We incorporated physical data as a prior on the interaction modes of an interaction variable in the factor graph. Some predictions are compatible with the evidence and some are not. We call the set of interaction modes compatible with the evidence *I^+^* and the set that is incompatible I^-^. For protein-DNA and phosphorylation data, where the protein of gene *A* binds the DNA of gene *B* or phosphorylates the product of gene *B*, *I^+^_AB_*={→, ─┤} and *I^-^_AB_* = {←,≠,=,├─}. For PPI data, *I^+^_AB_* ={→, ─┤,├─,=,←} and *I^-^_AB_* = {≠}. If there is no interaction data for *A* and *B*, then all interaction modes are in *I^+^_AB_* .We then define the evidence priors as *ρ_AB_(φ_AB_) =* 1 if *φ_AB_ ∈ I^+^* and *p*=10^-7^ otherwise. Varying the parameter *p* did not produce significantly different results.

Given the inferred log-likelihoods of each interaction mode *LL_AB_*(*φ_AB_*), we define the compatible log-likelihood as and the incompatible log-likelihood as . To compare pair-wise interaction predictions with physical evidence we defined the *margin of compatibility* (MOC) to be the difference in the log-likelihood of the most-likely interaction mode compatible with the evidence, LL^+^_AB_, relative to the most-likely mode incompatible with the evidence, LL^-^_AB_:

,

where values greater than zero indicate predictions compatible with the physical evidence, and negative values indicate predictions incompatible with the physical evidence.

**Pathway Expansion Attachment Estimation**

Using the notation of Markowetz *et al.* (2005), NEM scoring assigns a likelihood to a hidden attachment variable, *θ_e_*, for each E-gene *e*. The attachment variable can take on possible values {1, 2, ..., |S|}, recording the S-gene to which *e* is attached. To allow both inhibitory and stimulatory effects, we allow *θ_e_* to take on possible values ‑|*S*|,…,‑1,0,1,…,|*S*|, where the positive index *s* means *e* is activated by S‑gene *s* while *-s* means it is inhibited by *s*. The zero attachment state represents detachment from the entire signaling network, allowing *e*’s expression levels to be independent of any single S‑gene. Note that this possibility was not included in the original NEM framework, but a generalization has been described in Tresch *et al.* (2008) [8] in which effects can be attached to “null actions” that are analogous to including a detachment possibility introduced here. NEM model scoring assigns a likelihood to each possible value of θ*_e_*, which is used to find *e*’s attachment point.

**Artificial Networks Simulation**

We created synthetic signaling networks containing both inhibition and cycles to test FG-NEMs in a setting where the underlying network was known. We created a full underlying network for a set of S-genes, *T*, by connecting genes in *T* to each other by pair-wise interactions of varying strengths. Each interaction was associated with a strength chosen uniformly between 0.75 and 1. The strength of an interaction determines how well a signal passes from one S-gene to another, with 1 equal to perfect transmission and 0.75 equal to 25% transmission loss. An interaction was simulated to be inhibitory with probabilityλ. The parameter λ controls the proportion of inhibitory interactions between S-genes as well as inhibitory E-gene attachments. For example, λ=0 produces a network containing only activating S-gene connections and only activated E-gene attachments.

For each knock-down gene, *A*, quantized expression responses under Δ*A* were simulated by propagating a signal from *A* to the connected effect genes, keeping track of the sign and the strength of the signal. The final signal arriving at E-gene *e*, *s_e_*, was calculated as the product of the strengths and signs of all of the interactions on the path between *A* and *e*. E-gene *e* was then assigned to the activated (inhibited) distribution with probability |*s_e_|* for *s_e_*>0 (*s_e_*<0) and was assigned to the unaffected distribution with probability *(1-|s_e_|)*. E-genes chosen to be activated by *A* were given expression levels under Δ*A* that were less than their expression levels in wild-type. We refer to the distribution of expression changes of E-genes normally activated by *A* as the “activated distribution.” Similarly, we refer to the distribution of expression changes for repressed genes as the “inhibited distribution.” An E-gene *e*’s expression value under Δ*A* was generated by sampling from the activated, inhibited, or unaffected distributions depending on whether the path from *A* to *e* was inhibitory, stimulatory, or not connected (i.e. either *e* was not downstream of S or *e* was disconnected from the network completely). Replicated hybridizations were simulated by repeating the above procedure *R* times. To model incomplete knowledge of the network, a subset of S-genes was selected from *T* such that the resulting set of S-genes had size *k*|T|* for 0<*k*≤1.

The parameters λ, *k*, and *R* were varied for generating artificial networks. The number of E-genes per S-gene was set to 20 and cycles were always present in the network to model feedback loops that are often present in real biological networks. Unless otherwise noted, the number of S-genes in the network was sampled uniformly in the range of five to 15 and all S-genes were used in network recovery.

The E-gene expression distributions were modeled as Gaussians with unit variance and means equal to zero for the unaffected distribution, 1.75 for the inhibited distribution, and ‑1.75 for the activated distribution. Note that the activated distribution has a negative expression change on average since E-genes normally activated by a signal have lower expression levels relative to wild-type in knock-outs that block the activating signal*.* By the same reasoning, inhibited E-genes have positive expression changes relative to wild-type if a deletion blocks a normally inhibiting signal.

To estimate realistic levels of separation between the activated and inhibited distributions, we calculated the difference in mean expression of genes residing in pathways with somewhat opposing operations in the cell, such as subunits of the proteasome or ribosome. While the cell may turn on genes in both pathways, some conditions might favor protein expression over degradation (or vice versa). We reasoned that these conditions provide an estimate of the average difference in expression of an affected (up- or down-regulated) gene compared to an unaffected gene. We collected expression data for all of the genes in the proteasome and ribosome GO categories from a compendium of 3883 *H. sapiens*, 1049 *S. cerevisiae*, and 334 *D. melanogaster* microarray samples (see Figure S3). For each sample, a standardized absolute difference was calculated by computing the difference between the mean ribosome and proteasome levels and dividing the difference by the geometric mean of the respective standard deviations. The 95% quantile of the standardized absolute differences was found to be 1.75 and was used as the separation between the activated (or inhibited) distribution and the unaffected distribution for simulating E-gene expression changes. To avoid detecting differences in method performance due to fitting Gaussian parameters to simulated E-gene responses, the parameters for the expression factors in the FG-NEM and uFG-NEM were set to the same values used during simulation.

**Calculating Network Recovery Accuracy in Artificial Networks**

Each method’s ability to recover artificially generated S-gene networks using simulated E-gene data was measured. We simulated and predicted 500 networks, calculated the area under the precision-recall curve (AUC) for each predicted network, and recorded the mean and standard deviation of these AUCs.

The FG-NEM and uFG-NEM methods associate a likelihood score to each interaction mode for each pair of S-genes. Predicted networks for each method were produced by sorting the list of interactions by their mode preference scores and then keeping interactions with scores above a threshold. A prediction of an interaction between S-genes *A* and *B* was considered a true-positive if there was a direct or indirect path between *A* and *B* in the generated network having the same direction. For example, the prediction AB would be considered correct if the path ACB was present in the generated network and would not be considered correct if instead ACB was present. Note that because the top-scoring interaction mode for any pair was chosen, it is possible a method cannot reach a recall of 100% as an incorrect interaction will never be considered correct even for the most relaxed threshold. Precision was calculated as the fraction of true-positives out of all predicted positives, while recall was calculated as the fraction of true-positives out of all of gene pairs having a path in the generated network.

The area under the precision-recall curve (AUC) was calculated for each predicted network learned from artificial data. Predicted interactions were associated with their *mode preference score*, which was calculated by taking the log of the ratio of the likelihood of the top-scoring mode to the likelihood of the second best scoring mode A precision-recall curve was then generated for a predicted network by ranking its interactions by their *mode preference score* and calculating the precision and recall for a series of *score* cutoffs.

**Calculating Network Expansion Accuracy in Artificial Data**

To evaluate the ability of a method to predict new genes involved in a network, we performed a type of leave-one-out cross-validation. Each S-gene was held out in turn by removing the simulated expression data associated with its knock-down and then obtaining a LAR score for the S-gene in the recovered network. Each S-gene has simulated expression changes under the other S-gene knock-downs and thus the held-out S-gene can be scored like an E-gene. For a given pathway with a set of S-genes, each gene *s* was iteratively removed from the list of S-genes and included as one of the E-genes. In each iteration, the knockout data for *s* was deleted, resulting in the data set *X_-s_* representing the full matrix of expression for the pathway in which the columns (i.e. replicates) corresponding to the *s* knockout were removed. We used the percentiles of the LAR scores when comparing methods. The LAR percentile reflects how likely a held-out S-gene is attached to the network relative to all of the E-genes and is therefore more comparable across methods than using the LAR score directly.

The expression changes for the remaining S-genes were recorded in a reduced dataset *X_-s_*. We used an artificial network of 32 genes from which eight S-genes were randomly selected for simulated knock-down under which expression changes were simulated. We then ran FG-NEM and uFG-NEM on *X_-s_* and recorded the LAR percentile of *s*. We ran TM on the augmented matrix *[X­_-s_ : W]* where *W* was a matrix of the same size as *X_-s_* drawn from the no-change distribution. The percentile of *s* based on its correlation to an idealized vector was recorded.

**Calculating the Significance of Cancer Invasion Predictions**

To calculate the significance of S-gene interactions predicted for the invasiveness network, we permuted the second tier data 1000 times by shuffling the data within microarray hybridizations and then calculating the maximum likelihood interaction mode for each S-gene pair under every data permutation. An empirical *P* value was calculated independently for each S-gene pair as the fraction of likelihoods from permuted runs that exceed the likelihood of the non-permuted run. To calculate the significance of E-gene attachments, we permuted the data 1000 times by shuffling within gene rows, then predicting a network and calculating each E-gene’s LAR. An empirical *P*value for E-gene attachment was calculated independently for each E-gene as the fraction of LARs for that gene that exceed the LAR on the non-permuted data.

shRNA Construct Sequences

| Construct | Sense | Sequence |
| --- | --- | --- |
| MYO1G | sense | GATCCCCGGCCCTGAGTATGGCAAACTTCAAGAGAGTTTGCCATACTCAGGGCCTTTTTC |
|  | antisense | TCGAGAAAAAGGCCCTGAGTATGGCAAACTCTCTTGAAGTTTGCCATACTCAGGGCCGGG |
| BMPR1A | sense | GATCCCCGTGGCGGTGAAAGTATTCTTTCAAGAGAAGAATACTTTCACCGCCACTTTTTC |
|  | antisense | TCGAGAAAAAGTGGCGGTGAAAGTATTCTTCTCTTGAAAGAATACTTTCACCGCCACGGG |
| COLEC12 | sense | GATCCCCGCTGGACAGCCGGATAACTTTCAAGAGAAGTTATCCGGCTGTCCAGCTTTTTC |
|  | antisense | TCGAGAAAAAGCTGGACAGCCGGATAACTTCTCTTGAAAGTTATCCGGCTGTCCAGCGGG |
| AA099748 | sense | GATCCCCCTGTTCTCTCGTGTTTCAATTCAAGAGATTGAAACACGAGAGAACAGTTTTTC |
|  | antisense | TCGAGAAAAACTGTTCTCTCGTGTTTCAATCTCTTGAATTGAAACACGAGAGAACAGGGG |
| CAPN12 | sense | GATCCCCGATGATGCTGCAGGAATCCTTCAAGAGAGGATTCCTGCAGCATCATCTTTTTC |
|  | antisense | TCGAGAAAAAGATGATGCTGCAGGAATCCTCTCTTGAAGGATTCCTGCAGCATCATCGGG |
| scrambled | sense | GATCCCCCTCCAATGTGCTCGGTAATTTCAAGAGAATTACCGAGCACATTGGAGTTTTTC |
|  | antisense | TCGAGAAAAACTCCAATGTGCTCGGTAATTCTCTTGAAATTACCGAGCACATTGGAGGGG |

shRNA construct MYO1G targets the myosin 1G mRNA (GenBank accession number NM _033054). shRNA construct BMPR1A targets the bone morphogenetic protein receptor, type IA mRNA (NM_004329). shRNA construct COLEC12 targets the collectin sub-family member 12 mRNA (NM_130386). shRNA construct AA099748 targets an expressed sequence tag mRNA (AA099748). shRNA construct CAPN12 targets the calpain 12 mRNA (NM_144691). shRNA construct scrambled serves as a nonsense sequence negative control.

**References**

1. Markowetz F, Kostka D, Troyanskaya OG, Spang R (2007) Nested effects models for high-dimensional phenotyping screens. Bioinformatics 23: i305-312.

2. Frey BJ, Dueck D (2007) Clustering by passing messages between data points. Science 315: 972-976.

3. Mezard M, Parisi G, Zecchina R (2002) Analytic and algorithmic solution of random satisfiability problems. Science 297: 812-815.

4. Lee TI, Rinaldi NJ, Robert F, Odom DT, Bar-Joseph Z, et al. (2002) Transcriptional regulatory networks in Saccharomyces cerevisiae. Science 298: 799-804.

5. Ptacek J, Devgan G, Michaud G, Zhu H, Zhu X, et al. (2005) Global analysis of protein phosphorylation in yeast. Nature 438: 679-684.

6. Breitkreutz BJ, Stark C, Reguly T, Boucher L, Breitkreutz A, et al. (2008) The BioGRID Interaction Database: 2008 update. Nucleic Acids Res 36: D637-640.

7. Ashburner M, Ball CA, Blake JA, Botstein D, Butler H, et al. (2000) Gene ontology: tool for the unification of biology. The Gene Ontology Consortium. Nat Genet 25: 25-29.

8. Tresch A, Markowetz F (2008) Structure learning in Nested Effects Models. Stat Appl Genet Mol Biol 7: Article9.
